# Phylogeographic Clustering Suggests that Distinct Clades of Salmonella enterica Serovar Mississippi Are Endemic in Australia, the United Kingdom, and the United States

**DOI:** 10.1128/mSphere.00485-21

**Published:** 2021-09-22

**Authors:** R. A. Cheng, R. H. Orsi, M. Wiedmann

**Affiliations:** a Department of Food Science, Cornell University, Ithaca, New York, USA; Escola Paulista de Medicina/Universidade Federal de São Paulo

**Keywords:** *Salmonella*, phylogeography, polyphyly, prophage, whole-genome sequencing

## Abstract

Salmonella enterica serovar Mississippi is the 2nd and 14th leading cause of human clinical salmonellosis in the Australian island state of Tasmania and the United States, respectively. Despite its public health relevance, relatively little is known about this serovar. Comparison of whole-genome sequence (WGS) data of *S.* Mississippi isolates with WGS data for 317 additional S. enterica serovars placed one clade of *S.* Mississippi within S. enterica clade B (“clade B Mississippi”) and the other within section Typhi in S. enterica clade A (“clade A Mississippi”), suggesting that these clades evolved from different ancestors. Phylogenetic analysis of 364 *S.* Mississippi isolates from Australia, the United Kingdom, and the United States suggested that the isolates cluster geographically, with U.S. and Australian isolates representing different subclades (Ai and Aii, respectively) within clade A Mississippi and clade B isolates representing the predominant *S.* Mississippi isolates in the United Kingdom. Intraclade comparisons suggested that different mobile elements, some of which encode virulence factors, are responsible for the observed differences in gene content among isolates within these clades. Specifically, genetic differences among clade A isolates reflect differences in prophage contents, while differences among clade B isolates are due to the acquisition of a 47.1-kb integrative conjugative element (ICE). Phylogenies inferred from antigenic components (*fliC*, *fljB*, and O-antigen-processing genes) support that clade A and B Mississippi isolates acquired these loci from different ancestral serovars. Overall, these data support that different *S.* Mississippi phylogenetic clades are endemic in Australia, the United Kingdom, and the United States.

**IMPORTANCE** The number of known so-called “polyphyletic” serovars (i.e., phylogenetically distinct clades with the same O and H antigenic formulas) continues to increase as additional Salmonella isolates are sequenced. While serotyping remains a valuable tool for reporting and monitoring Salmonella, more discriminatory analyses for classifying polyphyletic serovars may improve surveillance efforts for these serovars, as we found that for *S.* Mississippi, distinct genotypes predominate at different geographic locations. Our results suggest that the acquisition of genes encoding O and H antigens from different ancestors led to the emergence of two Mississippi clades. Furthermore, our results suggest that different mobile elements contribute to the microevolution and diversification of isolates within these two clades, which has implications for the acquisition of novel adaptations, such as virulence factors.

## INTRODUCTION

The foodborne pathogen Salmonella enterica continues to incur a tremendous global disease burden, causing an estimated 88 million foodborne disease cases (95% uncertainty interval [UI], 34.7 million to 234.2 million) and 123,694 deaths (95% UI, 56,579 to 246,916) in 2010 (summarized from data in reference 1). Although the genus Salmonella includes just two species, S. bongori and S. enterica (2), at least 2,659 serological variants, called serovars, have been confirmed (3). Given this appreciable diversity, efforts to understand Salmonella pathogenesis have necessarily relied on the characterization of two model serovars, S. enterica serovars Typhi and Typhimurium, representing serovars that cause typhoidal salmonellosis and nontyphoidal salmonellosis (NTS), respectively.

Salmonella serovars are defined by the combination of somatic O (polysaccharide) and phase 1, phase 2, and sometimes phase 3 H (flagellar) antigens, encoded primarily by the *rfb* gene cluster (4) and *fliC* and *fljB*, respectively (5, 6). A total of 46 serogroups, which group serovars based on the presence of an O antigen that is considered characteristic of all serovars in that serogroup (7), have been defined (4). The observed structural variation among these O antigens, including the numbers and types of sugars as well as their linkage, is reflective of differences in the gene content in the O antigen gene cluster (4) as well as the horizontal acquisition of glycosyltransferases from bacteriophages (8) and plasmids (9). There are 114 known flagellar antigen types (6). Most serovars are biphasic (10), encoding and expressing two separate flagellar antigens (FliC and FljB), although monophasic serovars, such as S. Typhi (serotype I 9,12[Vi]:d:_) and S. Enteritidis (serotype I 1,9,12:g,m:_), and variants, such as the monophasic variant of S. Typhimurium (serotype I 4,[5],12:i:_), also exist. The acquisition of genes encoding different O and H antigens via horizontal gene transfer events facilitated by plasmids and bacteriophage (4, 11, 12) is considered to be the primary driver in the development of new and polyphyletic serovars.

S. enterica serovar Mississippi was first isolated from a stool sample collected from a food handler in the state of Mississippi in the United States and was confirmed as a new serovar in 1943 (13). *S.* Mississippi continues to be an important cause of human salmonellosis, particularly in the southern United States (14, 15) as well as Tasmania, Australia (16, 17). In the United States, *S.* Mississippi is the 14th most commonly isolated serovar from human clinical cases (18), while in Tasmania, *S.* Mississippi represents the 2nd most commonly isolated serovar, accounting for 37% of all nontyphoidal salmonellosis cases in that state (17). Although an environmental source or reservoir has not been definitively described for *S.* Mississippi, consumption of contaminated drinking water and contact with wild animals have been reported as risk factors for infection with *S.* Mississippi in Australia (16, 19), while in the United States, infection with *S.* Mississippi is associated with animal exposure (15). We previously suggested that *S.* Mississippi is likely a polyphyletic serovar (20, 21), although the overall population structure of isolates within this serovar was unknown. Therefore, we analyzed whole-genome sequence (WGS) data for *S.* Mississippi isolates from the United States, Australia, and the United Kingdom to better understand *S.* Mississippi populations in these locations.

## RESULTS

### WGS data confirm the presence of two main polyphyletic clades of *S.* Mississippi.

**FIG 1:**
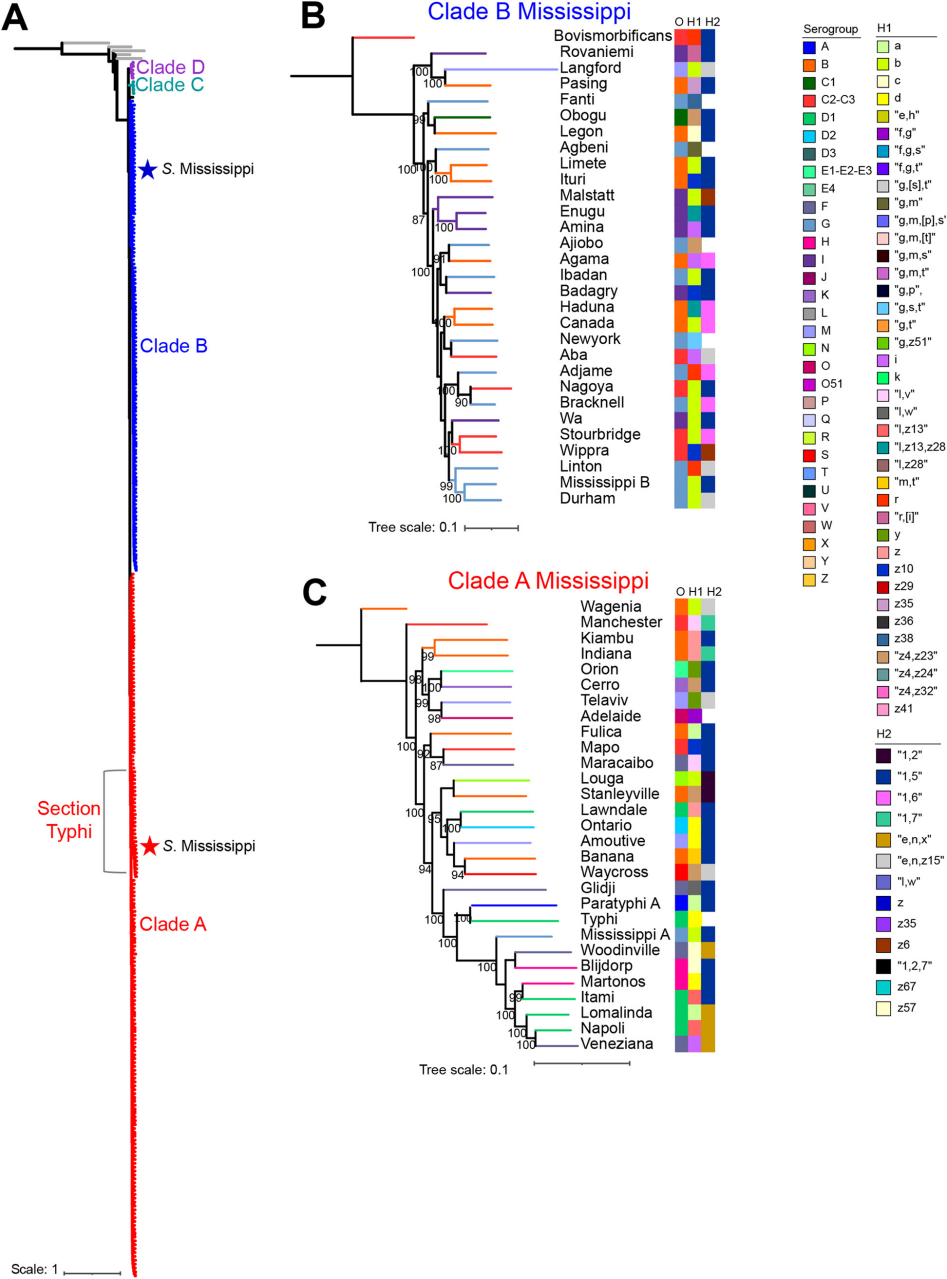
*S.* Mississippi isolates cluster within S. enterica subsp. *enterica* clades A and B. (A) Phylogeny inferred from maximum likelihood analysis of 10,905 core SNPs compared across 318 unique serovars, including representative isolates of each *S.* Mississippi clade (denoted with red [clade A, section Typhi] and blue [clade B] stars). A total of 100 bootstrap repetitions were performed. Branches are color-coded to reflect the phylogenetic clade of the serovar. The tree is rooted by S. enterica subsp. *arizonae* (GenBank assembly accession number GCA_000018625), which has been used previously as an outgroup for S. enterica (22). (B and C) Core SNPs for serovars that clustered with clade B Mississippi (29 serovars; 57,090 core SNPs) (B) and clade A Mississippi (29 serovars; 61,144 core SNPs) (C) were identified, and phylogenetic trees were inferred. The bootstrap values listed represent the averages from 1,000 bootstrap repetitions. Colored strips on the right show the serogroup (rightmost strip) and phase 1 (H1) and phase 2 (H2) flagellar antigens (middle and leftmost strips, respectively) reported for a given serovar. Some serovars do not encode a phase 2 flagellar antigen, and therefore, these serovars lack a colored square to signify that they are monophasic. Outgroups were selected based on the phylogenetic analyses in panel A and were *S.* Wagenia and *S.* Bovismorbificans for clades A and B, respectively.

Previous studies characterizing the typhoid toxin in NTS serovars suggested that the typhoid toxin islet was restricted to certain 7-gene multilocus sequence typing (MLST) sequence types of *S.* Mississippi. These *S*. Mississippi sequence types clustered separately in phylogenetic analyses containing other serovars, suggesting that *S*. Mississippi was likely polyphyletic (20, 21). To confirm the polyphyly of *S.* Mississippi, we inferred a core single-nucleotide polymorphism (SNP)-based phylogeny of two *S.* Mississippi isolates and representative isolates for 317 additional serovars, including five additional S. enterica subspecies. We hypothesized that *S.* Mississippi may share a most recent common ancestor (MRCA) with other serogroup O13 serovars, and therefore, one isolate for all serogroup O13 serovars having publicly available WGS data was included in this data set (28 out of a total of 69 S. enterica subsp. *enterica* O13 serovars). This phylogenetic analysis confirmed the presence of two distinct *S.* Mississippi clades (Fig. 1A), designated clade A and B Mississippi here, reflective of their sharing MRCAs with serovars in S. enterica subsp. *enterica* clades A and B (21, 22).

To assess the evolutionary history of these two Mississippi clades, we next assessed the serovars with which these Mississippi clades share an MRCA. Clade B Mississippi shared an MRCA with Salmonella serovar Durham (serotype I 13,23:b:e,n,z_15_), and *S*. Mississippi and *S*. Durham shared an MRCA with Salmonella serovar Linton (serotype I 13,23:r:e,n,z_15_) (Fig. 1B). Interestingly, *S.* Ibadan, which differs slightly in its O antigen (i.e., *S*. Ibadan encodes O 13,22 versus 1,13,23 in *S*. Mississippi [the “1” antigen is due to prophage-mediated glucosylation]) but has the same H1 and H2 antigens as *S.* Mississippi (7), is more closely related to Salmonella serovars Badagry (serotype I 16:z_10_:1,5), Ajiobo (serotype I 13,23:z_4_,z_23_:_), and Agama (serotype I 4,12:i:1,6) (Fig. 1B). Approximately one-third of the serovars in this section of clade B belong to serogroup O13, and 38% of the serovars encode the “b” variant of the H1 antigen (Fig. 1B). The “1,5” H2 antigen was encoded by nearly one-half of the serovars in this clade, although it is worth noting that nearly one-third of the serovars in our overall data set (representing 319 total genomes, including representatives of the two Mississippi clades) have this H2 antigen, suggesting that it is very common among S. enterica subsp. *enterica* serovars in general (see Data Set S1 in the supplemental material).

10.1128/mSphere.00485-21.6 DATA SET S1 *In silico* prediction of serotype for all isolates included in phylogenetic analyses. Download Data Set S1, XLSX file, 0.1 MB. Copyright © 2021 Cheng et al. 2021 Cheng et al. https://creativecommons.org/licenses/by/4.0/ This content is distributed under the terms of the Creative Commons Attribution 4.0 International license.

Clade A Mississippi shares an MRCA with Salmonella serovars Woodinville, Blijdorp, Martonos, Itami, Lomalinda, Veneziana, and Napoli (Fig. 1C), placing this clade within section Typhi, a subset of S. enterica subsp. *enterica* clade A (22). Within section Typhi, which included 29 serovars in the data set used here (Fig. 1C), *S.* Mississippi is the only serogroup O13 serovar and is just one of three serovars having the “b” H1 antigen (Fig. 1C). Finally, the H2 antigen “1,5” was also common among serovars in this subclade (57% encoded this antigen).

Overall, these data confirm that *S.* Mississippi represents two distinct clades within S. enterica subsp. *enterica* clade B and section Typhi (within clade A).

### Clade A Mississippi is primarily isolated from the United States and Australia, while clade B Mississippi contains isolates from the United Kingdom.

**FIG 2:**
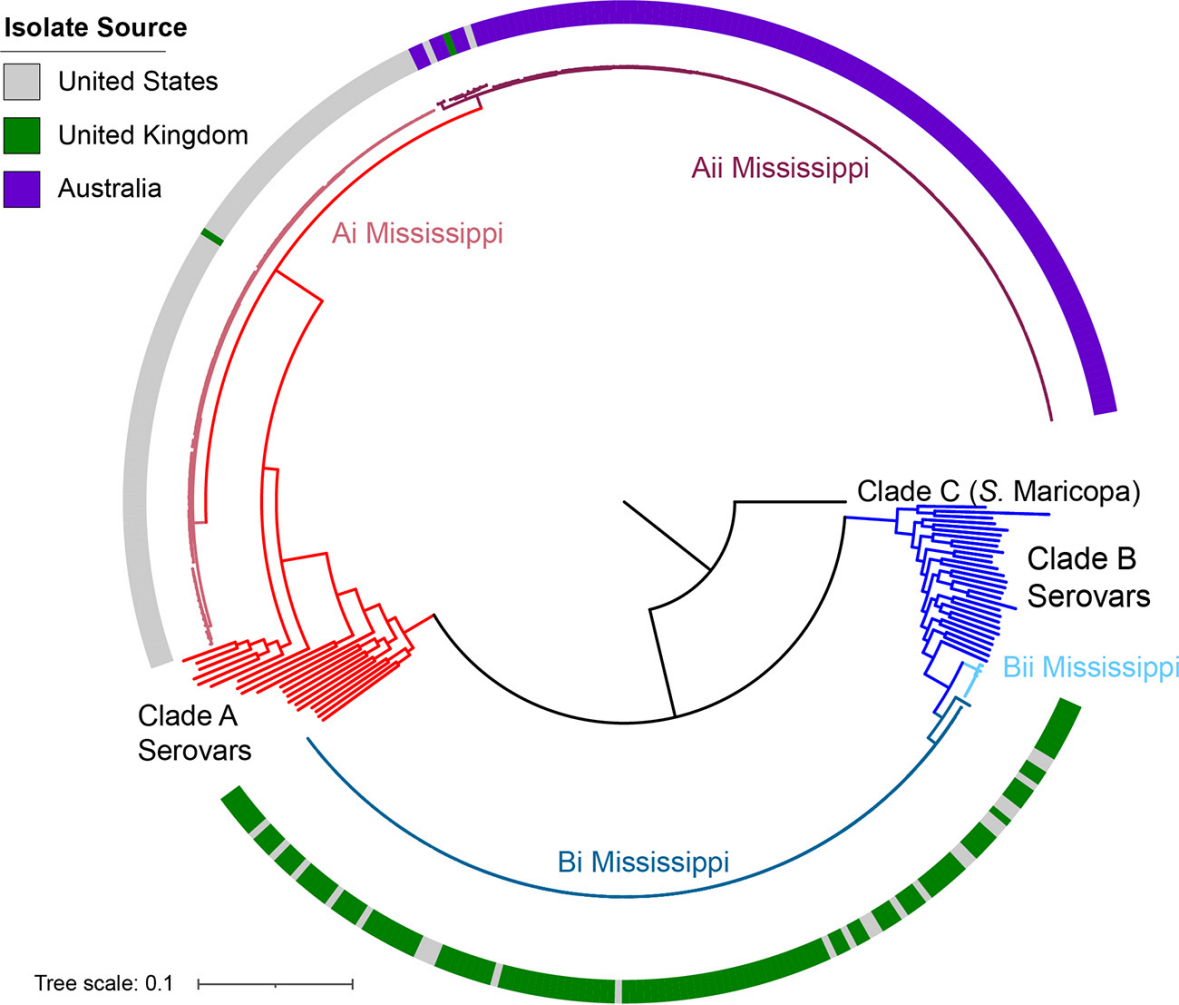
Phylogeographic clustering of *S.* Mississippi clades suggests that different *S.* Mississippi isolates are endemic in Australia, the United Kingdom, and the United States. A phylogeny was inferred from maximum likelihood analysis of 54,880 core SNPs among 364 *S.* Mississippi isolates and representative isolates of serovars that share an ancestor with clade A and B Mississippi as determined from Fig. 1B and C. A total of 500 bootstrap repetitions were performed, and the tree was rooted with the clade C serovar *S.* Maricopa as the outgroup. The colored squares shown external to the tree are colored to reflect the country of isolation.

We next curated a database of *S.* Mississippi genomes to assess the phylogenetic relatedness of isolates within clade A and B Mississippi. Given that >95% of assemblies available (accessed 7 October 2019) were from Australia, the United Kingdom, or the United States, we focused our analyses on *S.* Mississippi originating from these three locations (see Data Set S2 for a complete list of metadata for these isolates). Among 364 *S*. Mississippi assemblies, 223 were assigned to clade A, and the remaining 141 isolates were assigned to clade B (Fig. 2A and B). Within each clade, two major subclades were identified based on tree topology and core SNP distances. The distribution of the geographical location of the isolates varied significantly by phylogenetic clade (*P* = 0.0005 by Fisher’s exact test). Clade Ai included 98 isolates from the United States and 1 isolate from the United Kingdom. Clade Aii included all 121 isolates from Australia as well as 1 and 2 isolates from the United Kingdom and the United States, respectively. In contrast, clade Bi was composed of isolates from the United Kingdom (109 UK isolates versus 25 U.S. isolates), and clade Bii included just 7 isolates, all from the United Kingdom (Fig. 2A and B). Together, these data suggest that different subtypes of *S.* Mississippi are endemic in Australia, the United Kingdom, and the United States.

10.1128/mSphere.00485-21.7 DATA SET S2 Metadata for all *S.* Mississippi isolates used in this analysis. Download Data Set S2, XLSX file, 0.05 MB. Copyright © 2021 Cheng et al. 2021 Cheng et al. https://creativecommons.org/licenses/by/4.0/ This content is distributed under the terms of the Creative Commons Attribution 4.0 International license.

### Diversification of clade Ai and Aii Mississippi is reflected by differences in prophage contents.

**FIG 3:**
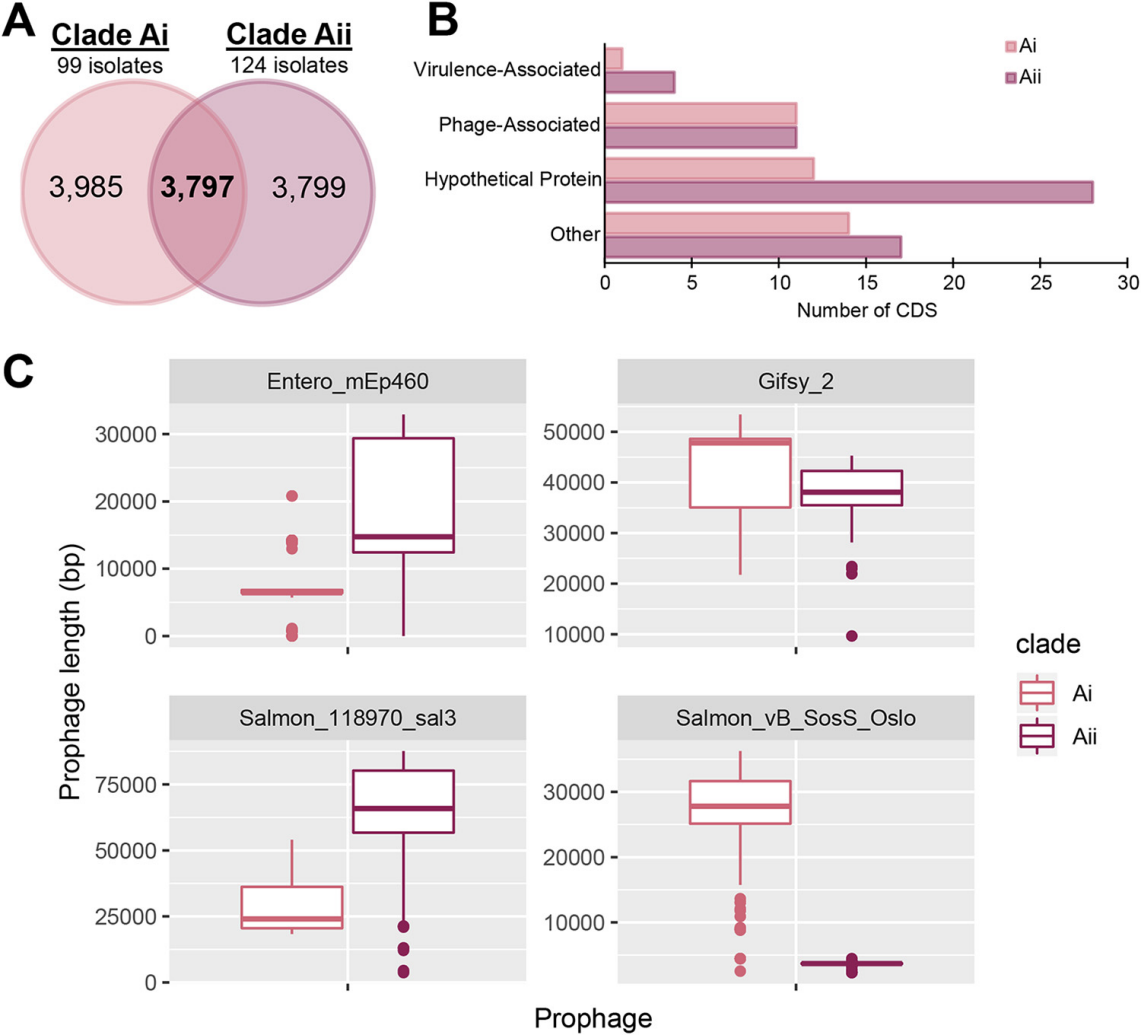
Diversification of clade Ai and Aii isolates is mediated by the acquisition and loss of prophages. (A) Comparison of core genes (present in at least 99% of isolates in the comparison) among clade Ai and Aii isolates as well as genes shared by isolates in both clades. (B) Categories of genes that were present in at least 90% of isolates in one clade but that were absent from all isolates in the other clade. Genes were categorized manually into each group based on annotation suggested by InterPro and/or Prokka. Genes in the phage-associated category were annotated as encoding phage components (such as tail fibers and capsid proteins, etc.) or integration-related machinery necessary for prophage insertion (such as integrase and recombinase, etc.). Hypothetical proteins represent genes that did not have any annotation suggested by InterPro, while genes in the “other” category represent genes with annotations suggesting that they were associated with nonvirulence and nonphage functions. A full list of all genes and their annotations can be found in Data Set S3 and Table S1 in the supplemental material. CDS, coding DNA sequences. (C) Box plot summaries of the nucleotide lengths of prophages Entero_mEp460, Gifsy-2, Salmon_118970_sal3, and Salmon_vB_SosS_Oslo for all isolates in clade Ai (*n* = 99 isolates) and clade Aii (*n* = 124 isolates). Nucleotide lengths were summed from hits for each local BLAST alignment for each prophage.

To better assess differences among the clade Ai and Aii Mississippi isolates, we calculated the core genomes and pangenomes of isolates in these clades to characterize the mechanisms driving their diversification. Clade Ai and Aii Mississippi isolates differed by an average of 5,239 core SNPs (range, 5,026 to 6,345 SNPs; total of 18,151 core SNPs in the analysis). Within each clade, core SNP differences ranged from 0 to 1,821 (median, 735) and 2 to 2,672 (median, 173) for clade Ai and Aii isolates, respectively. The core genome (defined here as genes present in ≥99% of genomes) of clade Ai isolates included 3,985 genes, compared to 3,799 genes considered core to clade Aii isolates (Fig. 3A), while the pangenome of clade Aii isolates (6,167 genes) was 744 genes larger than the pangenome of clade Ai isolates (5,423 genes).

10.1128/mSphere.00485-21.2 TABLE S1 InterPro identification of genes among clade Ai and Aii isolates that were present in 90% of isolates in one clade but completely absent from all isolates in the other clade. Download Table S1, DOCX file, 0.03 MB. Copyright © 2021 Cheng et al. 2021 Cheng et al. https://creativecommons.org/licenses/by/4.0/ This content is distributed under the terms of the Creative Commons Attribution 4.0 International license.

10.1128/mSphere.00485-21.8 DATA SET S3 Gene presence/absence data for Ai versus Aii isolates and Bi versus Bii isolates. Download Data Set S3, XLSX file, 0.2 MB. Copyright © 2021 Cheng et al. 2021 Cheng et al. https://creativecommons.org/licenses/by/4.0/ This content is distributed under the terms of the Creative Commons Attribution 4.0 International license.

To assess specific differences in gene contents, we identified genes that were significantly overrepresented in Ai and Aii isolates. These analyses identified a total of 1,182 genes that were significantly overrepresented (Benjamini-Hochberg-corrected *P* value of <0.01) (Data Set S3) among Ai or Aii isolates. Thirty-eight genes were detected in >90% of Ai isolates but were absent from all Aii isolates (Fig. 3B). A number of these genes were annotated as phage associated (e.g., genes annotated as “phage tail collar domain,” “bacteriophage P22,” and “anti-RecBCD protein 2”); therefore, we binned terms based on their InterPro-assigned functional family (Table S1). Roughly one-third of the terms were phage associated, and the remaining two-thirds were hypothetical proteins (*n* = 12) or represented a different cellular process (*n* = 14) (Fig. 3B). Only one of the genes that were associated with Ai genomes was predicted to be associated with virulence; this gene (detected in 97 out of 99 Ai genomes) encoded an NF-κB-p65-degrading zinc protease and was mapped to a genomic region identified by Phaster as the prophage Gifsy-1.

A total of 60 genes were detected in >90% of Aii isolates but were absent from all Ai isolates. Roughly half of these genes (*n* = 28) represented hypothetical proteins, 11 were phage associated, 17 were associated with a different cellular process, and 4 were virulence associated (Fig. 3B), including typhoid toxin genes (*cdtB*, *pltA*, and *pltB*) and *sopE*, encoding the guanine nucleotide exchange factor SopE.

As genes in the phage-associated category represented 22% of genes that were differentially present among isolates in both clades, we first used Phaster to identify prophages among a subset of 5 isolates in each clade. Overall, 20 different putative prophages were identified among this subset of isolates (Table S2), which were then used to select four prophage genomes that were differentially present in clade Ai versus clade Aii isolates for performing BLAST alignments among all 223 clade Ai and Aii genomes. Of the four prophages queried, Gifsy-2 and Salmon_vB_SosS_Oslo had a larger median nucleotide length in clade Ai (47.8 kb and 27.8 kb, respectively) than in clade Aii (38.1 kb and 3.7 kb, respectively) genomes, while phages Entero_mEp460 and Salmon_118970_sal3 had a larger median nucleotide length in clade Aii (14.8 kb and 65.9 kb, respectively) genomes than in clade Ai (6.4 kb and 24.1 kb, respectively) genomes. Together, these results suggest that differences in prophage gene contents are largely driven by the presence of different prophages in clade Ai and Aii genomes.

10.1128/mSphere.00485-21.3 TABLE S2 Summary of Phaster results for representative clade Ai and Aii genomes. Download Table S2, DOCX file, 0.03 MB. Copyright © 2021 Cheng et al. 2021 Cheng et al. https://creativecommons.org/licenses/by/4.0/ This content is distributed under the terms of the Creative Commons Attribution 4.0 International license.

Overall, these results suggest that while clades Ai and Aii share a MRCA, the two clades have since diverged, accumulating >5,000 core SNPs, with gene acquisition/loss events resulting primarily from the gain or loss of prophages.

### Clade Bii isolates encode a 47.1-kb ICE that is absent in all clade Bi isolates.

**FIG 4:**
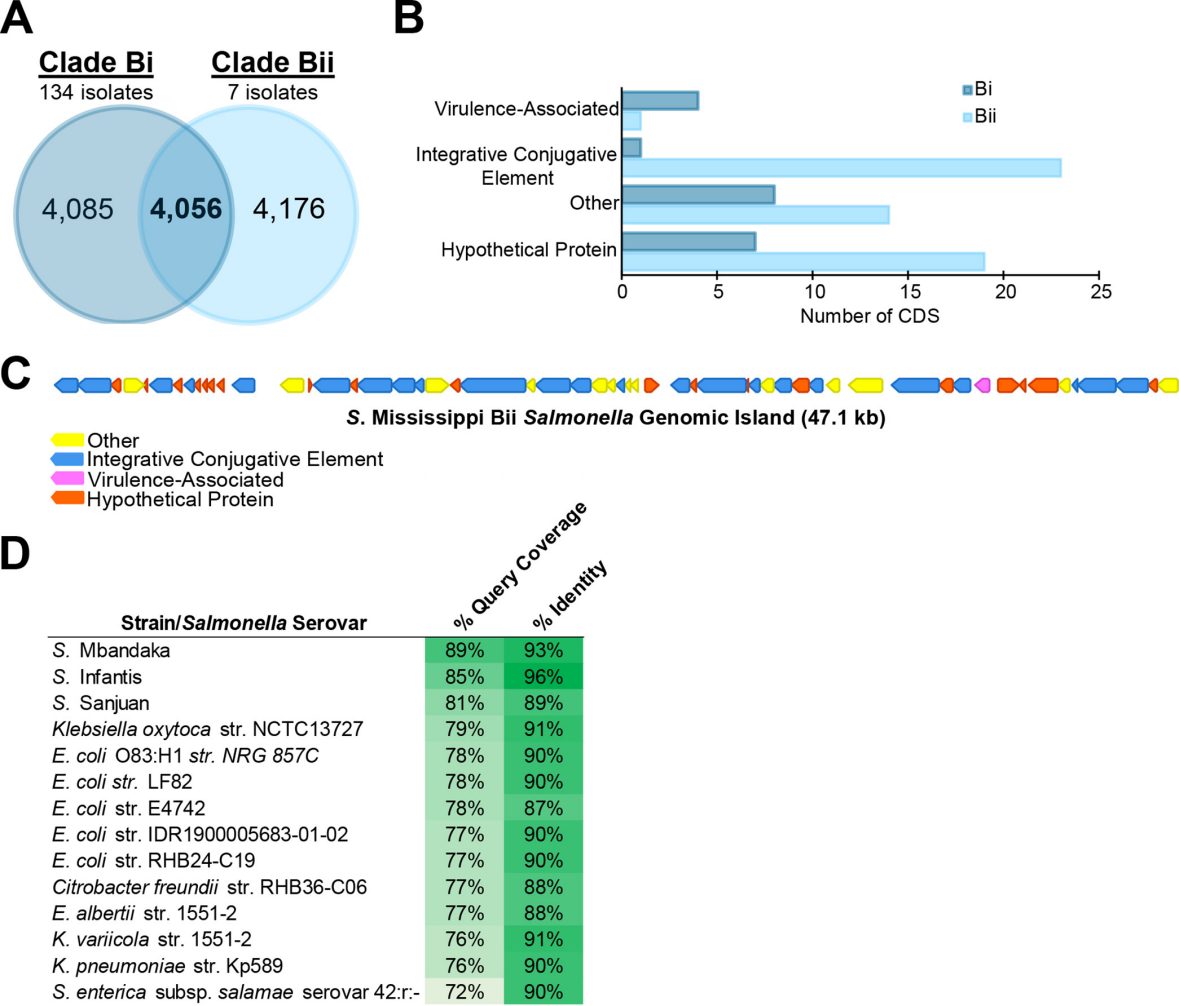
Differences in gene contents of clade Bi and Bii Mississippi isolates are mediated by the acquisition of a 47.1-kb integrative conjugation element by clade Bii Mississippi. (A) Comparison of core genes (present in at least 99% of isolates in the comparison) among clade Bi and Bii isolates as well as genes shared by isolates in both clades. (B) Categories of genes that were present in at least 90% of isolates in one clade but that were absent from all isolates in the other clade. Genes were categorized manually into each group based on annotation suggested by InterPro and/or Prokka. Genes in the integrative conjugative element category were annotated as genes associated with integration or conjugative transfer (such as integrases and Tra proteins, etc.). Hypothetical proteins represent genes that did not have any annotation suggested by InterPro, while genes in the “other” category represent genes with annotation suggesting that they were associated with nonvirulence and nonphage functions. See Data Set S3 and Table S3 in the supplemental material for a full list of all genes and their annotations. (C) Organization and annotation of genes identified in panel B that were located within the 47.1-kb integrative conjugative element found in all 7 clade Bii Mississippi isolates. (D) Results of a discontiguous BLAST search for the clade Bii ICE in other Salmonella isolates and other bacteria. Only hits with >70% query coverage are shown. *E. albertii*, Escherichia albertii; *K. variicola*, Klebsiella variicola.

Next, we conducted analyses for clade Bi and Bii isolates to assess the genetic diversity within and among these Mississippi subclades. Clade Bi isolates (*n* = 134 isolates; predominantly UK isolates) and clade Bii isolates (*n* = 7; all UK isolates) differed by an average of 6,210 core SNPs (range, 6,088 to 7,064 core SNPs; 11,062 total core SNPs in comparison). Within each clade, the numbers of core SNPs ranged from 0 to 4,806 (median, 64) and from 16 to 916 (median, 399) for Bi and Bii isolates, respectively. A total of 4,056 genes were core (i.e., present in ≥99% of genomes) (Fig. 4A) to isolates in clades Bi and Bii. Clade Bi isolates shared 4,085 core genes and had a pangenome size of 5,232 genes (Fig. 4A). The pangenome of clade Bii isolates was smaller and included 4,347 genes, with 4,176 considered core to Bii isolates (Fig. 4A).

10.1128/mSphere.00485-21.4 TABLE S3 InterPro identification of genes among clade Bi and Bii isolates that were present in 90% of isolates in one clade but completely absent from all isolates in the other clade. Download Table S3, DOCX file, 0.02 MB. Copyright © 2021 Cheng et al. 2021 Cheng et al. https://creativecommons.org/licenses/by/4.0/ This content is distributed under the terms of the Creative Commons Attribution 4.0 International license.

Among a total of 20 genes that were detected in ≥90% of isolates in clade Bi but absent from clade Bii, most were associated with either hypothetical proteins (*n* = 7) or other cellular functions (*n* = 8) (see Table S3 for a full list of all genes). The remaining genes were annotated as transposases (classified as integrative conjugative elements [ICEs] in Fig. 4B) or were virulence associated (*n* = 4), including three genes (*yraJ*, *yraI*, and *yehB*; homologs of genes in the Lpf and Stc fimbrial gene clusters in Salmonella) associated with chaperone-usher fimbria assembly and *sopD2* encoding a secreted effector that blocks lysosome fusion with the Salmonella-containing vacuole during intracellular infection of host cells (23). Among 57 genes that were present in at least 90% of clade Bii isolates and absent from all clade Bi isolates, roughly half were annotated as integration or conjugation associated (*n* = 23), while the remaining genes represented hypothetical proteins (*n* = 19), genes with other predicted functions (*n* = 14), and one virulence factor (Fig. 4B). Mapping of the integration- and conjugation-associated genes showed that these genes represented a 47.1-kb ICE within a contig (nearly 500,000 bp long), which included several Tra genes and integrases as well as one gene encoding the fimbrial subunit SbaA (homolog of YadA in Escherichia coli) (Fig. 4C and Data Set S3); none of the ICE-associated genes were detected in the clade Bi assemblies, suggesting that this element was most likely acquired by clade Bii isolates after divergence from clade Bi. Using discontiguous BLAST analysis, we queried other bacterial genomes to assess how prevalent this ICE was among other Salmonella serovars and other bacterial genera. The ICE was identified in several other Salmonella serovars (>70% query coverage), including S. enterica subsp. *enterica* serovars Mbandaka (clade A), Infantis (clade A), and Sanjuan (clade C) and one S. enterica subsp. *salamae* serovar (Fig. 4D). Additionally, the ICE was found in other bacteria, including select strains of Escherichia coli, Klebsiella oxytoca, Klebsiella pneumoniae, and Citrobacter freundii (Fig. 4D). Together, clade Bi and Bii Mississippi, despite sharing a common geographic source (United Kingdom) and MRCA, are separated by >6,000 core SNPs, with differences in gene contents largely reflecting the presence of a 47.1-kb ICE in clade Bii Mississippi.

### Clade A and B Mississippi *fliC* genes, which encode phase 1 flagellin, represent two distinct sequence types acquired from different ancestral serovars.

Next, we characterized the genes encoding surface antigens to assess how clade A and B Mississippi evolved to display the same serotype. Flagellar antigens are thought to be primarily acquired via horizontal gene transfer (12). Initial attempts to extract the full-length 1,488-bp *fliC* sequence were unsuccessful for over 20% of the isolates, and therefore, we extracted and compared SNPs within a 717-bp (239 codons) internal sequence. The 717-bp internal *fliC* sequence was conserved, with just 9 polymorphic sites detected among the 364 *S.* Mississippi isolates. A total of 4 haplotypes were detected: (i) haplotype I included 215 clade Ai and Aii Mississippi isolates; (ii) haplotype II included 7 Mississippi clade Aii isolates, which differed from haplotype I by 1 SNP; (iii) haplotype III included one clade Ai isolate, which differed from haplotype I by 1 SNP; and (iv) haplotype IV included all 141 clade Bi and Bii isolates, which differed from haplotypes I, II, and III by 2, 3, and 2 SNPs, respectively. Overall, this indicates that *fliC* is conserved at the clade level, with clade Ai/Aii and Bi/Bii isolates having distinct *fliC* sequences.

**FIG 5:**
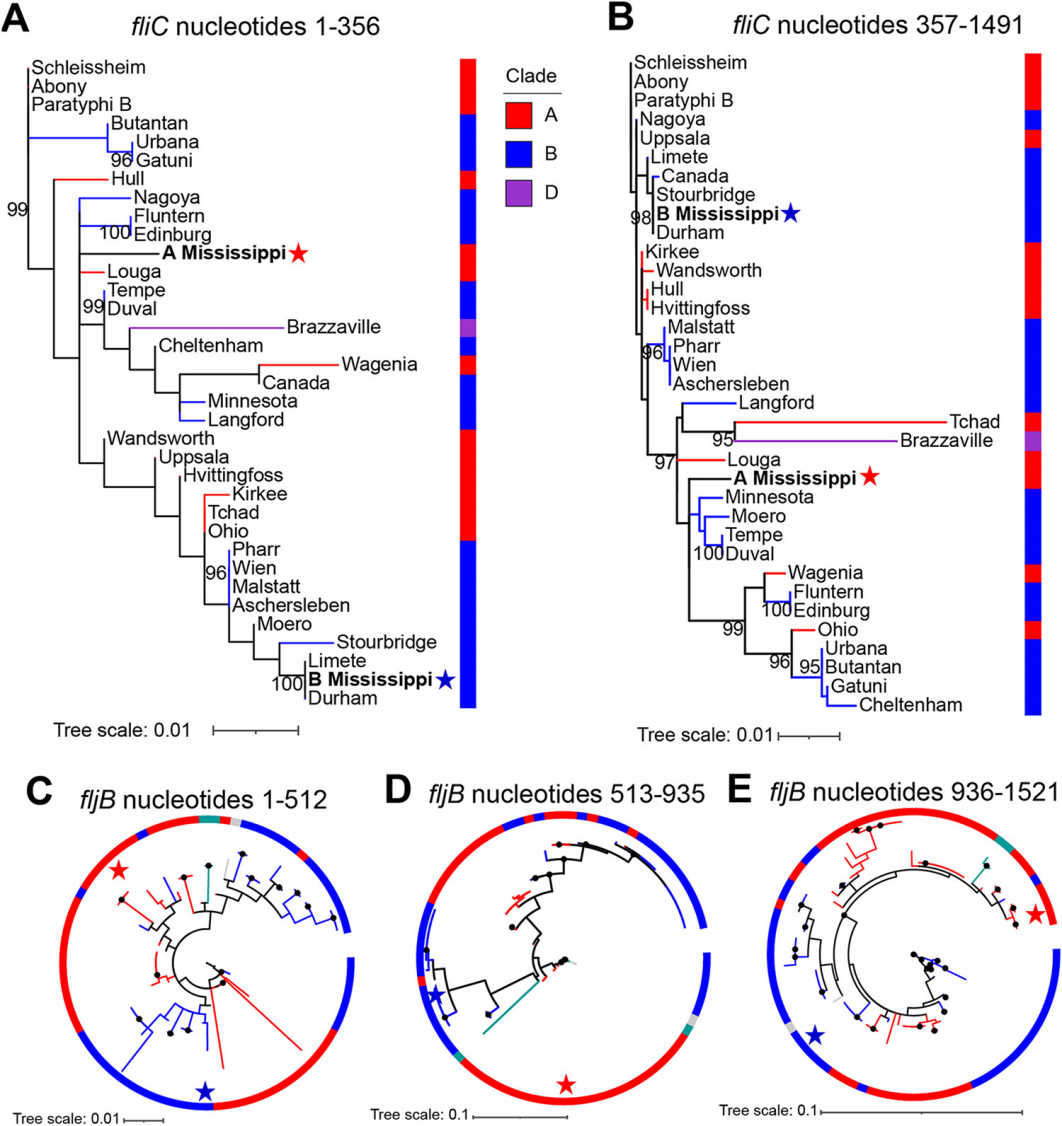
Phylogenies inferred for *fliC* and *fljB* support the acquisition of these genes from different ancestors for clade A and B Mississippi. (A and B) Maximum likelihood inference of *fliC* before (A) and after (B) a recombination breakpoint at nt 356 to 357 detected by GARD for representative isolates of clade A and B Mississippi and 33 additional serovars. (C to E) Maximum likelihood inference for *fljB* segments representing nucleotides 1 to 512 (C), 513 to 935 (D), and 936 to 1521 (E) for representative isolates of clade A and B Mississippi and 80 additional serovars. Branches are color-coded to reflect the phylogenetic clade of the serovar. Stars represent the branches corresponding to clade A (red stars) and clade B (blue stars) *S.* Mississippi *fljB* sequences. Phylogenies were inferred with IQ-TREE with 1,000 ultrafast bootstrap repetitions. Values shown on branches represent ultrafast bootstrap approximations; values are shown only for branches with >95% bootstrap support.

Given that the internal *fliC* sequence was largely conserved among Ai/Aii and Bi/Bii Mississippi isolates, we next selected representative strains of haplotypes I (representing the majority of clade Ai and Aii Mississippi) and IV (representing clade Bi and Bii Mississippi *fliC*) for which a full-length *fliC* sequence could be extracted and compared these sequences with *fliC* sequences from 33 additional serovars from other S. enterica phylogenetic clades (Fig. 5A). The full-length *fliC* sequences from clade A and B Mississippi differed by 25 polymorphic sites. Clade B Mississippi *fliC* shared 100% nucleotide identity with *fliC* from *S*. Durham; these two serovars share an MRCA based on core SNPs (Fig. 1B). Clade A Mississippi *fliC* represented a unique haplotype; serovars with the fewest SNP (17 SNPs) distances from clade A Mississippi *fliC* included clade B Salmonella serovars Duval and Tempe and clade A section Typhi serovar Louga.

Analysis with the genetic algorithm for recombination detection (GARD) detected a single recombination breakpoint in *fliC*, between nucleotides (nt) 356 and 357, and we therefore inferred phylogenies of *fliC* for nucleotide positions 1 to 356 and 357 to 1491 (*S*. Tchad *fliC* is 1,491 nucleotides) to assess whether an ancestor(s) could be inferred for *fliC* acquisition in clade A and B Mississippi isolates. In both phylogenies (nucleotides 1 to 356 and 357 to 1491), *fliC* from clade B Mississippi formed a monophyletic clade with those from other clade B serovars (*S*. Limete, *S*. Stourbridge, and *S*. Durham) (Fig. 5A and B), suggesting that it acquired *fliC* from an ancestral clade B serovar. In contrast, clade A Mississippi’s *fliC* represents a distinct branch with low bootstrap support in both phylogenies and with *fliC* from different serovars for each segment of *fliC*, suggesting the acquisition of *fliC* from a serovar not included in our analysis. Overall, these analyses suggest that clade A and B Mississippi acquired their *fliC* genes from different ancestral serovars.

### The phase 2 flagellin gene *fljB* also represents two distinct haplotypes for clade A and B Mississippi, suggesting the acquisition of this flagellar antigen from different donors.

Next, we applied the same analyses to the variable region (nt 514 to 1263; amino acids [aa] 172 to 421) within the flagellar type 2 antigen encoded by *fljB* type “1,5” to first assess the conservation of this gene among isolates within clade A and B Mississippi. Among the 364 *S.* Mississippi isolates, we identified just two haplotypes each, representing all clade A (Ai and Aii) or all clade B (Bi and Bii) Mississippi isolates (i.e., *fljB* from each clade shared 100% nucleotide identity with all other *S*. Mississippi strains in the clade). These two *fljB* haplotypes differed by 30 SNPs.

We next compared these two *S*. Mississippi *fljB* haplotypes with full-length *fljB* sequences from 80 additional S. enterica subsp. *enterica* serovars. Although clade A and B Mississippi *fljB* genes represented unique haplotype sequences (i.e., they did not share 100% nucleotide identity with *fljB* from any other serovar), clade A Mississippi *fljB* differed by 2 SNPs from the clade A section Typhi serovar Blijdorp, and clade B Mississippi differed by 3 SNPs from clade B serovar Pasing. Two recombination breakpoints were detected within *fljB*, and we therefore inferred phylogenies for the three nucleotide segments (nt 1 to 512, 513 to 935, and 936 to 1521) (Fig. 5C to E). For all three phylogenies, clade B Mississippi *fljB* clustered with those of the other clade B serovars (Fig. 5C to E). For segments spanning nucleotides 513 to 935 and 936 to 1521, clade B Mississippi *fljB* formed a monophyletic clade with clade B serovars Pasing (serotype I 4,12:z_35_:1,5), Enugu (serotype I 16:l,[z_13_],z_28_:[1,5]), Nagoya (serotype I 6,8:b:1,5), and Amina (serotype I 16:i:1,5). The observed phylogenetic clustering of *S*. Mississippi *fljB* with those of other clade B serovars for all three segments suggests that clade B Mississippi likely acquired its *fljB* from an ancestral clade B serovar, although further phylogenetic analyses will be important for assessing which specific segments were acquired vertically versus horizontally.

Clade A Mississippi *fljB* clustered with *fljB* from section Typhi serovar Blijdorp, with which it also shares a common ancestor (Fig. 1C), across all three gene segments, although it should be noted that low bootstrap support was observed for the first gene segment (nucleotides 1 to 512). In addition, section Typhi serovars Lawndale and Ontario clustered with clade A Mississippi and *S*. Blijdorp in the phylogeny inferred for nucleotides 936 to 1521; while *S*. Lawndale and *S*. Ontario cluster within section Typhi, they do not share a MRCA with clade A Mississippi (Fig. 1C). This suggests that clade A Mississippi *fljB* nucleotides 936 to 1521 were most likely acquired from a common ancestor shared with Salmonella serovars Ontario and Lawndale. However, for nucleotides 1 to 935, based on the results here, we can infer that this part of *fljB* was most likely acquired from another clade A serovar, based on the placement of clade A Mississippi within a clade of *fljB* from predominantly clade A serovars, but due to low bootstrap support, an exact donor could not be determined.

Overall, these analyses suggest that recombination of *fljB* with different serovars in clades A and B represents the most likely acquisition of this locus in clade A and B Mississippi, respectively.

### Clade A and B Mississippi likely acquired O-antigen genes from different ancestors in their respective phylogenetic clades.

**FIG 6:**
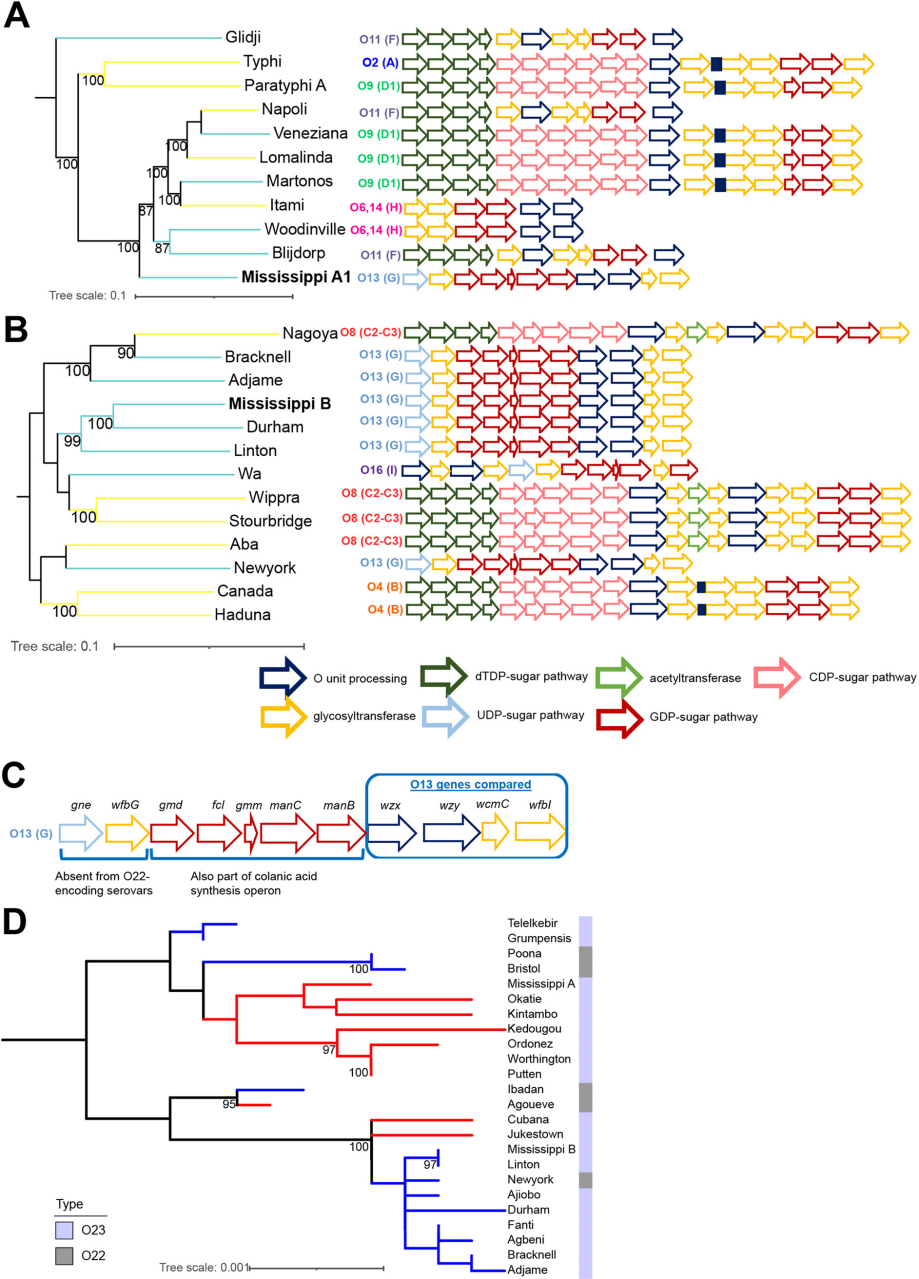
Comparison of Mississippi A and B serogroup O13 (G) O antigen gene clusters suggests acquisition from different ancestors. (A and B) O-antigen gene clusters established previously (4) were mapped onto the phylogenetic trees shown in Fig. 1B and C for clade A (A) and clade B (B) *S*. Mississippi. Genes are color-coded based on their predicted function as described previously by Liu and colleagues (4). Branches represent O antigens that are initiated with either galactose (Gal initiated) (yellow) or *N*-acetylglucosamine/*N*-acetylgalactosamine (GlcNAc/GalNAc initiated) (teal). (C) O-antigen gene cluster for serogroup O13(G) as described previously by Liu and colleagues (4). Only *wzx*, *wzy*, *wcmC*, and *wfbI* were compared here as *gne* and *wfbG* were missing from most of the serovars that also react with O22 antisera, and *gmd*, *fcl*, *gmm*, *manC*, and *manB* are also present in the colanic acid synthesis operon; therefore, sequences for these genes could not be reliably extracted for all isolates or serovars examined. (D) Concatenated sequences for *wzx*, *wzy*, *wcmC*, and *wfbI* were used to infer a phylogeny for 24 serovars, including representative strains of clade A and B *S.* Mississippi. Branches are colored based on the phylogenetic clade of the serovar determined in Fig. 1 (red, clade A; blue, clade B). Bootstrap values of >95% are listed at the nodes (1,000 ultrafast bootstrap repetitions were performed). Colored bars represent the O type (O22 or O23) listed in the serovars’ antigenic formulas. The tree is midpoint rooted.

Finally, we examined the genes encoding the O antigen to assess potential routes of acquisition. The O antigens produced by Salmonella constitute two general classes based on whether the first sugar in the O unit is galactose (Gal) or *N*-acetylgalactosamine (GalNAc)/*N*-acetylglucosamine (GlcNAc) (4). To this end, we first mapped the O sugar type (i.e., Gal initiated versus GlcNAc/GalNAc initiated) and *rfb* locus (4) for the clade A and B serovars sharing common ancestors with clade A and B *S.* Mississippi (Fig. 6A). While some serovars had a similar initiating sugar as serovars with which they shared a MRCA, we also saw evidence of serovars that did not share an O antigen with the other serovars in the subclade. Clade A Mississippi isolates cluster with several other serovars with GlcNAc/GalNAc-initiated O antigens; however, the O-antigen operon structures of those serovars are very different (Fig. 6A). Clade B Mississippi shares a common ancestor with five other serovars with O13 (Fig. 6A), suggesting that the acquisition of the O-antigen-processing genes for clade B Mississippi was likely from a common ancestor shared with clade B Salmonella serovars Bracknell, Adjame, Durham, Linton, and Wa. As genes encoding the O antigen are thought to be inherited as a complete unit (11), we hypothesized that comparison of the nucleotide sequences of the genes within the O13-encoding operon may provide additional clues to the origin of the O antigen for clade A and B Mississippi.

The operon encoding O13 has been reported previously (4, 24). We first sought to confirm the presence of all 11 genes for the O-antigen-processing gene cluster in representative isolates for clade A and B Mississippi. However, as multiple genes involved in the GDP-sugar pathway are present in both the colanic acid operon and the O-antigen synthesis gene cluster (i.e., *gmd*, *fcl*, *gmm*, *manC*, and *manB*), these genes were excluded from our SNP analysis as the short-read sequence data used here could not be confidently mapped to one locus over the other (Fig. 6B). Likewise, as we did not detect *gne* and *wfbG* (which are located directly upstream of colanic acid synthesis genes) in the majority of the O13 serogroup serovars that also react with the O22 antisera, suggesting that these genes may not be essential for the O13 serotype but may be helpful for differentiating between O22 and O23 serotypes, we excluded *gne* and *wfbG* from our analyses. We therefore compared the gene sequences of *wzx*, *wzy*, *wcmC*, and *wfbI* for isolates representing 22 additional serovars (9 clade A serovars and 13 clade B serovars) with 1 isolate each to represent clade A and B Mississippi, as preliminary analyses suggested that these genes were highly conserved across isolates within clades A (Ai and Aii) and B (Bi and Bii) (0 to 3 SNPs per gene) (Table S4). Clade B Mississippi isolates had identical nucleotide sequences for all four genes with clade B serovar Linton (Fig. 6C and Table 1); in addition, some of these genes also shared 100% nucleotide identity with O-antigen genes from other clade B serovars (Table 1). On the other hand, clade A Mississippi isolates had unique nucleotide sequences of O-antigen genes with the exception of *wcmC*; 15 additional serovars, including both S. enterica subsp. *enterica* clade A and B serovars, had an identical *wcmC* allele (Table 1). It is important to note that *wcmC* was also the least diverse, having just 6 polymorphic sites detected across all 24 serovars and *S.* Mississippi clades. Genes *wzx* and *wzy* were the most diverse among these 24 serovars and *S.* Mississippi clades, with 22 and 25 polymorphic sites, respectively (Table 1). A maximum likelihood phylogeny inferred from concatenated sequences of *wzx*, *wzy*, *wcmC*, and *wfbI* further supported that for clade B Mississippi, the O-antigen-processing gene cluster was acquired vertically from a common ancestor, while the O-antigen-processing genes for clade A Mississippi likely resulted from horizontal acquisition, although additional analyses with a greater number of serovars will be necessary to ascertain whether this event happened in an ancestral serovar not included in our analysis or occurred with the diversification of this Mississippi clade from its MRCA. Overall, these analyses support that the O-antigen-processing genes were acquired from different ancestors for clade A and B Mississippi.

**TABLE 1:** Comparison of O-antigen genes *wzx*, *wzy*, *wcmC*, and *wfbI* among representative O13 serovars

Gene	No. of haplotypes	No. of polymorphic sites	Salmonella serovar(s)	
			Genomes in clade A Mississippi haplotype^*a*^	Genomes in clade B Mississippi haplotype^*b*^
*wcmC*	7	6	Agoueve, Ajiobo, Bracknell, Cubana, Fanti, Grumpensis, Ibadan, Kintambo, Linton, Mississippi B, Newyork, Okatie, Ordonez, Putten, Telelkebir, Worthington	Agoueve, Ajiobo, Bracknell, Cubana, Fanti, Grumpensis, Ibadan, Kintambo, Linton, Newyork, Okatie, Ordonez, Putten, Telelkebir, Worthington
*wfbI*	9	13		Adjame, Agbeni, Ajiobo, Bracknell, Cubana, Durham, Fanti, Grumpensis, Linton, Newyork, Okatie, Telelkebir
*wzx*	14	22		Agbeni, Durham, Fanti, Jukestown, Linton, Newyork
*wzy*	16	25		Linton

a List of all serovars that shared 100% nucleotide identity with the respective Mississippi clade A genes.

b List of all serovars that shared 100% nucleotide identity with the respective Mississippi clade B genes.

10.1128/mSphere.00485-21.5 TABLE S4 Summary of BLASTn searches for O-antigen genes *wcmC*, *wfbI*, *wzx*, and *wzy*. Download Table S4, DOCX file, 0.01 MB. Copyright © 2021 Cheng et al. 2021 Cheng et al. https://creativecommons.org/licenses/by/4.0/ This content is distributed under the terms of the Creative Commons Attribution 4.0 International license.

## DISCUSSION

In this study, we describe the diversity of the geographically associated clades of *S.* Mississippi, which evolved from two separate MRCAs from serovars in clade B and clade A section Typhi. While infections with *S.* Mississippi are common in regions in the United States (14, 18) and Australia (16, 17), there were few reports about *S.* Mississippi in the United Kingdom (25). Gene presence/absence comparisons suggested that differentiation among isolates within the major clades is driven by the acquisition/loss of mobile elements such as prophage (clades Ai and Aii) and integration/conjugation elements (clade Bii). Finally, our analyses suggest that clade A and B Mississippi independently acquired the genes encoding their somatic and flagellar antigens from different ancestors in their respective phylogenetic clades.

### *S.* Mississippi isolates form separate clades within S. enterica subsp. *enterica* clades A and B.

While a number of Salmonella serovars (e.g., *S.* Kentucky [26], *S.* Derby [27], *S.* Newport [28], and others [21, 22, 29]) have been described as being polyphyletic, it is rare for these polyphyletic serovars to span multiple S. enterica subsp. *enterica* clades, as was observed in this study for *S.* Mississippi, which spans S. enterica subsp. *enterica* phylogenetic clades A and B. In a recent analysis of 247 S. enterica subsp. *enterica* serovars, 24 were confirmed to be polyphyletic (22). Interestingly, only Salmonella serovars I 47:z_4_,z_23_:–, Kisarawe, and Montevideo included isolates that spanned A and B S. enterica subsp. *enterica* clades; the remaining 21 polyphyletic serovars had clades within the same S. enterica subsp. *enterica* clade, but some serovars included isolates that spanned different subclades within S. enterica subsp. *enterica* clade A (22). The observation that relatively few polyphyletic serovars span multiple S. enterica subsp. *enterica* clades (e.g., clades A and B) may reflect the fact that the majority of characterizations often include only a few isolates from each serovar, which is likely insufficient to assess the population structure of a serovar. As the availability of WGS data for understudied serovars continues to increase, it is likely that the number of polyphyletic serovars will also increase.

Polyphyletic serovars have been associated previously with adaptation to specific hosts. For example, different lineages of the polyphyletic serovar *S.* Derby are associated with poultry and swine hosts, suggesting that isolates from these different lineages possess unique adaptations that allow them to preferentially colonize/infect certain hosts (27). Similarly, some clades of the polyphyletic serovar *S.* Montevideo are associated with cattle, while other *S.* Montevideo clades include isolates from a broader range of hosts (30). In a recent phylogenetic study characterizing *S.* Mississippi isolates from Australia, human clinical isolates clustered closely with isolates from livestock (e.g., ovine, avian, caprine, and bovine), water, and domestic (e.g., feline, canine, and alpaca) and wild (e.g., platypus, lizard, kangaroo, and wombat) animals, suggesting that clade Aii Mississippi isolates have a broad host range (16). Conversely, only three nonhuman *S.* Mississippi isolates were classified into clades Ai, Bi, and Bii; these isolates were from a horse, a dog, and an unidentified environmental source (all clade Ai) (see Data Set S2 in the supplemental material). In the United States, isolation of *S.* Mississippi from horses represents the most common isolation source for nonhuman clinical *S.* Mississippi isolates (representing 36% of all reported *S.* Mississippi isolates from nonhuman clinical sources between 1968 and 2011), suggesting that this serovar may also be an important cause of salmonellosis in horses (31). Although it is tempting to hypothesize that the low number of nonhuman clinical isolates from clade B suggests that isolates within these clades are human adapted, sampling and phylogenetic comparisons of *S.* Mississippi from nonhuman sources will be necessary to better understand any host adaptations that may exist among isolates in clade B.

### Populations of *S.* Mississippi from Australia, the United Kingdom, and the United States represent distinct phylogenetic clades.

Phylogenetic analyses of select bacterial pathogens have suggested that some genotypes show a region-specific distribution pattern, while others are distributed more broadly across multiple countries/continents. For example, multidrug-resistant S. Typhi H58 demonstrates phylogeographic clustering, with one lineage predominating in Southeast Asia and the other lineage predominating in Africa and southern Asia (32). Similar phylogeographic relationships have been observed among other Salmonella serovars isolated from different geographic locations within the same country, including S. Typhimurium (33), *S.* Cerro (34), and *S.* Dublin (35), as well as between countries (e.g., *S.* Kentucky [36], *S.* Dublin [37], S. Typhi [38], and S. Typhimurium [39]). Ford and colleagues (16) also observed that *S.* Mississippi isolates from New Zealand (excluded from our analyses) represented a distinct phylogenetic clade separate from all Australian isolates, suggesting that there are likely additional phylogenetic clades of *S.* Mississippi outside those characterized here. Similarly, clade Bii *S.* Mississippi, which contained just 7 isolates from the United Kingdom, may also represent a distinct, geographically isolated clade representing a different region within the United Kingdom.

We also identified differences in the virulence factors encoded among isolates in different phylogeographic clades, some of which were associated with the presence/absence of prophage. Genes encoding typhoid toxin and the guanine exchange factor SopE were detected among all isolates in clade Aii but were absent from isolates in clade Ai. While the contributions of these virulence factors have been demonstrated at the cellular level, their role in human clinical salmonellosis is not as straightforward. For example, human challenge models failed to identify a role for typhoid toxin in acute typhoid fever as volunteers infected with a toxin-null strain had disease presentation indistinguishable from that of volunteers infected with the wild-type strain (40). Furthermore, analyses have shown that the percentages of *S.* Mississippi infections that result in invasive disease are similar in the United States (0.5% of cases are invasive [41]) and Australia (2.6% cases are invasive [17]), where clade Ai and Aii Mississippi represent the predominant clades, respectively. Future studies that combine epidemiological surveillance data with associated patient metadata will be beneficial for furthering our understanding of how these factors may influence disease severity among Ai and Aii isolates.

Overall, the strong geographical association observed for different *S.* Mississippi clades provides important and practically relevant information that may support source-tracking investigations of *S.* Mississippi clinical cases or food contamination events, as has been suggested previously for other Salmonella serovars and foodborne pathogens (42, 43).

### The acquisition of flagellar and O-antigen-processing genes from different donors led to two distinct clades of *S.* Mississippi.

Not surprisingly, our analyses suggest that *S.* Mississippi clades A and B are the result of the acquisition of flagellar and O-antigen-processing genes from different ancestral serovars. This is consistent with multiple studies that have suggested that the most parsimonious explanation for the observed diversity of flagellin and O-antigen-processing genes is that these antigens are the result of multiple horizontal gene transfer events (11, 12, 44). For example, the evolution of *S*. Lubbock (serotype I 6,7:g,m,s:e,n,z_15_) from an MRCA shared with *S.* Mbandaka (serotype I 6,7,14:z_10_:e,n,z_15_) (45), and of *S*. Sendai (serotype I 1,9,12:a:1,5) from an MRCA shared with *S*. Paratyphi A (serotype I 1,2,12:a:[1,5]) (46), is proposed to have resulted from the horizontal acquisition of *fliC*. Our data, on the other hand, suggest that for clade B Mississippi (serotype I 1,13,23:b:1,5), the acquisition of part of *fljB* may have accompanied its divergence from *S.* Durham (I 13,23:b:e,n,z_15_), although additional analyses will be necessary to determine the directionality of this event. Our analyses of *fliC*, *fljB*, and some of the O-antigen-processing genes suggested that clade B Mississippi acquired these genetic loci from ancestral clade B serovars. The evolution of clade A Mississippi, however, is considerably less clear and suggests that additional serovars within section Typhi remain to be characterized, as clade A Mississippi is currently the only known serovar within section Typhi with the O13 antigen, and comparisons of *fliC* and *fljB* with sequences from other serovars revealed an obvious donor.

## MATERIALS AND METHODS

### Non-Mississippi S. enterica isolates.

An isolate set of 318 unique serovars was compiled for assessing the population structure of *S.* Mississippi in the context of S. enterica. Isolates represent serovars that (i) are commonly isolated from human clinical infections in the United States (18), (ii) belong to serogroup O13 (7), (iii) were used in population analyses performed previously (22), and (iv) have antigenic formulas that include FliC antigen “b” or FljB antigen “1,5” (7). Additional isolates representing S. enterica subsp. *salamae*, *arizonae*, *diarizonae*, *houtenae*, and *indica* were also included in the final data set (see Data Set S1 in the supplemental material).

### Selection of *S*. Mississippi whole-genome sequence data.

As of 7 October 2019, whole-genome sequence data for 383 isolates listed as *S*. Mississippi were available in the NCBI Pathogen Detection database (https://www.ncbi.nlm.nih.gov/pathogens/). Assemblies and raw sequence data for a total of 358 *S.* Mississippi isolates were downloaded after excluding isolates that (i) were not sequenced on an Illumina platform; (ii) did not list a valid collection date (i.e., the year was not provided); (iii) were not from Australia, the United Kingdom, or the United States; and (iv) had assemblies with >300 contigs. An additional 6 isolates of *S.* Mississippi that had been sequenced previously by our laboratory were also included in the data set (Data Set S2).

### Genome assembly, quality assessment, and *in silico* serotype prediction.

Assemblies were downloaded from the NCBI using a custom Perl script, and the remaining isolates were assembled with SKESA version 2.4.0 (47) using data from the NCBI Sequence Read Archive database. Contigs of <200 bp were removed, and quality was assessed with QUAST version 4.0 (48). The Salmonella *In Silico* Typing Resource (SISTR) was used to confirm the reported serotype of the isolate (49).

### Reference-free single-nucleotide polymorphism analyses.

kSNP3 version 3.1 (50) was used to identify core SNPs using kmer sizes (ranging from 15 to 19) suggested by kchooser. Maximum likelihood trees based on core SNP matrices obtained from kSNP3 were inferred using RAxML version 8.2.12 (51) with the substitution model GTRGAMMAX (general time reversible with gamma model rate of heterogeneity) and the Lewis ascertainment bias correction.

### Assignment of core genomes and pangenomes.

Prokka version 1.12 was used to annotate *S.* Mississippi genomes using the Gram neg option (52). Core genomes and pangenomes were calculated with Panaroo (53). Scoary version 1.6.14 was used to perform associations and statistical analyses to identify core and accessory genes (54). Open reading frames were annotated with InterProScan version 5.44-79.0 with the iprlookup option (55).

### Mining of phage-associated genes in clade Ai and Aii assemblies.

Phaster (56) was used to search for prophages in clade Ai and Aii assemblies (5 assemblies each) having the lowest number of contigs among all assemblies in these clades. Four prophages were then selected for a local BLAST search among all 223 clade A genomes. The prophages (GenBank accession numbers) used in the BLAST analysis were Entero_mEp460 (NC_019716.1), Salmon_vB_SosS_Oslo (NC_018279.1), Salmon_118970_sal3 (NC_031940.1), and Gifsy-2 (NC_010393.1).

### Detection of recombination within *fliC* and *fljB* internal sequences.

Recombination was assessed using the genetic algorithm for recombination detection (GARD) as part of the HyPhy package v.2.5.15 (57) in the interactive command line mode with the general discrete (GDD) option for site variation with a rate class equal to 3.

### Comparison of *fliC* and *fljB* sequences in *S.* Mississippi isolates.

Initial attempts to extract full-length sequences of *fliC* and *fljB* were unsuccessful because these genes were not fully assembled for approximately half of the clade B Mississippi genomes used here. This problem is due to the genetic similarity of the conserved regions found in these two genes, which are difficult to assemble using short sequencing reads and low sequencing depth (i.e., genomes sequenced with shorter reads and/or with low average coverage were less likely to present fully assembled *fliC* and *fljB* sequences). We first used BBmap version 38.73 (58) to align raw reads to full-length sequences of *fliC* and *fljB* for a select set of *S.* Mississippi isolates and then used Geneious software version 11.1.5 to visualize the mapping results. These analyses showed that the coverage at the 5′ and 3′ ends for both *fliC* and *fljB* was roughly 2-fold higher than the coverage in the middle of the gene (Fig. S1), suggesting that because of the conservation of the flagellar antigen at the 5′ and 3′ ends, full-length sequences for these two loci could not be extracted for all sequences. We therefore first extracted and compared internal sequences for *fliC* (nt 514 to 1230) and *fljB* (nt 514 to 1263) using BLASTn. For comparisons of *fliC* and *fljB* with those of other serovars, we extracted full-length sequences (33 and 80 sequences for comparisons of *fliC* and *fljB*, respectively) and compared these with representative sequences from clade A and B Mississippi isolates (Sequence Read Archive accession numbers SRR2969498 and SRR5812039, respectively). Phylogenetic trees were inferred using IQ-TREE v.2.0.7 (59), using the mpi option to select the substitution model with the best fit.

10.1128/mSphere.00485-21.1 FIG S1 Variable regions of *fliC* and *fljB* used in phylogenetic comparisons. (A and B) Raw reads were mapped to full-length *fliC* (A) and *fljB* (B) for representative isolates (results from a single isolate are shown), with coverage represented by the height of the histograms at each nucleotide position. The length of the variable fragment used in comparisons is shown by the red bracket. (C and D) Comparison of the numbers and positions of SNPs present in the conserved regions of *fliC* and *fljB* for both the 5′ (C) and 3′ (D) regions illustrating the conservation of the flagellin at these termini. Green bars represent conserved nucleotide positions, and white bars represent SNPs. Tracks represent both the nucleotide (top colored track for each gene) and the amino acid (bottom colored track for each gene) coding sequences. Download FIG S1, TIF file, 1.8 MB. Copyright © 2021 Cheng et al. 2021 Cheng et al. https://creativecommons.org/licenses/by/4.0/ This content is distributed under the terms of the Creative Commons Attribution 4.0 International license.

### Comparison of O-antigen cluster genes.

O-antigen cluster genes for O13 were defined previously (4). As several genes encoding O13 are also located in the colanic acid synthesis pathway (i.e., *gmd*, *fcl*, *gmm*, *manC*, and *manB*), which is directly upstream of the O-antigen gene cluster, and initial BLAST searches suggested few SNPs in these genes, we limited our analyses to *wzx*, *wzy*, *wcmC*, and *wfbI* as these genes were present in all O13 serovars examined here. Nucleotide sequences were extracted with BLAST, and alignments and phylogenetic analyses of concatenated gene sequences were performed as described above.

### Data availability.

All data generated in this study are included in the supplemental material. All sequence data are available in the NCBI database. Any additional raw data will be shared upon request.
