# Supplementary material for: Phylogeographic Clustering Suggests that Distinct Clades of Salmonella enterica Serovar Mississippi Are Endemic in Australia, the United Kingdom, and the United States
Source: mSphere. 2021 Sep 22;6(5):e00485-21. doi: 10.1128/mSphere.00485-21 (PMC8550085; doi:10.1128/mSphere.00485-21)
Supplement: TABLE S1 [file msphere.00485-21-st001.docx]

| **Gene*^a^*** | **InterPro*^b^*** | **IPR No.*^b,c^*** | **Category*^d^*** | **No. Ai Genomes with Gene** | **No. Aii Genomes with Gene** |
| --- | --- | --- | --- | --- | --- |
| group_1873 | Homeobox-like domain superfamily | IPR009057 | Other | 99 | 0 |
| group_1461 | - | - | Hypothetical Protein | 99 | 0 |
| group_1391 | Protein of unknown function DUF977 | IPR010382 | Other | 99 | 0 |
| group_2913 | Helicase HerA, central domain | IPR002789 | Other | 99 | 0 |
| group_2734 | Bacteriophage P4, Psu superfamily | IPR038395 | Phage-Associated | 99 | 0 |
| group_306 | - | - | Hypothetical Protein | 99 | 0 |
| group_2302 | DNA primase/nucleoside triphosphatase, C-terminal | IPR004968 | Phage-Associated | 99 | 0 |
| esiB_2 | Sel1-like repeat | IPR006597 | Other | 99 | 0 |
| group_930 | Helicase, superfamily 3, DNA virus | IPR014015 | Phage-Associated | 99 | 0 |
| intA_4 | DNA breaking-rejoining enzyme, catalytic core | IPR011010 | Phage-Associated | 99 | 0 |
| dnaC_2/3/4 | DNA replication protein DnaC/insertion sequence putative ATP-binding protein | IPR028350 | Other | 99 | 0 |
| group_2355 | Hok/gef cell toxic protein | IPR000021 | Other | 99 | 0 |
| group_2802 | - | - | Hypothetical Protein | 99 | 0 |
| group_3162 | - | - | Hypothetical Protein | 99 | 0 |
| group_586 | - | - | Hypothetical Protein | 99 | 0 |
| cfaE | CblD-like pilus biogenesis initiator | IPR010888 | Other | 99 | 0 |
| group_1848 | - | - | Hypothetical Protein | 99 | 0 |
| group_2583 | - | - | Hypothetical Protein | 98 | 0 |
| group_377 | NFkB-p65-degrading zinc protease | IPR025208 | Virulence-Associated | 97 | 0 |
| group_2826 | RecT family | IPR018330 | Other | 97 | 0 |
| group_2635 | Ead/Ea22-like protein | IPR025153 | Phage-Associated | 97 | 0 |
| group_20 | Exodeoxyribonuclease 8 | IPR010584 | Other | 97 | 0 |
| group_2705 | AP2/ERF domain | IPR001471 | Other | 97 | 0 |
| group_274 | SIR2-like domain | IPR039444 | Other | 97 | 0 |
| group_2650 | Integrase, catalytic core | IPR001584 | Phage-Associated | 96 | 0 |
| group_59 | Phage tail collar domain | IPR011083 | Phage-Associated | 96 | 0 |
| group_2910 | - | - | Hypothetical Protein | 96 | 0 |
| group_2364 | - | - | Hypothetical Protein | 96 | 0 |
| group_2613 | DNA breaking-rejoining enzyme, catalytic core | IPR011010 | Phage-Associated | 94 | 0 |
| group_1746 | Bacteriophage P22, anti-RecBCD protein 2 | IPR020500 | Phage-Associated | 91 | 0 |
| group_2312 | Protein of unknown function DUF2737 | IPR020295 | Other | 91 | 0 |
| group_1436 | - | - | Hypothetical Protein | 91 | 0 |
| group_2893 | Domain of unknown function DUF550 | IPR007538 | Other | 90 | 0 |
| group_2046 | - | - | Hypothetical Protein | 90 | 0 |
| group_1699 | - | - | Hypothetical Protein | 90 | 0 |
| group_2697 | RNA polymerase sigma factor 70, region 4 type 2 | IPR013249 | Other | 90 | 0 |
| group_2699 | Domain of unknown function DUF551 | IPR007539 | Phage-Associated | 89 | 0 |
| group_2804 | Ead/Ea22-like protein | IPR025153 | Phage-Associated | 89 | 0 |
| group_2399 | - | - | Hypothetical Protein | 0 | 124 |
| esiB_1/hcpA/hcpC | - | IPR006597 | Other | 0 | 124 |
| group_1259 | Homeobox-like domain superfamily | IPR009057 | Other | 0 | 124 |
| group_1241 | - | - | Hypothetical Protein | 0 | 124 |
| group_2719 | - | - | Hypothetical Protein | 0 | 124 |
| group_1649 | - | - | Hypothetical Protein | 0 | 124 |
| group_2320 | Protein of unknown function DUF1493 | IPR010862 | Other | 0 | 124 |
| cdtB | Cytolethal distending toxin B | IPR003539 | Virulence-Associated | 0 | 122 |
| group_977 | Lysozyme-like domain superfamily | IPR023346 | Other | 0 | 122 |
| ptxA | Bordetella pertussis toxin A | IPR003898 | Virulence-Associated | 0 | 122 |
| group_2831 | DNA breaking-rejoining enzyme, catalytic core | IPR011010 | Phage-Associated | 0 | 122 |
| group_3168 | Host-nuclease inhibitor Gam | IPR009274 | Phage-Associated | 0 | 122 |
| group_3169 | - | - | Hypothetical Protein | 0 | 122 |
| group_2692 | Enterotoxin | IPR008992 | Virulence-Associated | 0 | 122 |
| group_1859 | - | - | Hypothetical Protein | 0 | 122 |
| group_1450 | Campylobacter phage CGC-2007, Cje0229 | IPR010767 | Phage-Associated | 0 | 122 |
| group_2664 | - | - | Hypothetical Protein | 0 | 121 |
| group_2460 | - | - | Hypothetical Protein | 0 | 121 |
| group_2064 | Domain of unknown function DUF4224 | IPR025319 | Other | 0 | 120 |
| group_2499 | Bacteriophage lambda, Bet | IPR010183 | Phage-Associated | 0 | 120 |
| group_2207 | Protein of unknown function DUF1482 | IPR009954 | Other | 0 | 120 |
| group_3376 | - | - | Hypothetical Protein | 0 | 120 |
| group_2191 | - | - | Hypothetical Protein | 0 | 120 |
| group_1850 | - | - | Hypothetical Protein | 0 | 120 |
| group_3287 | Exonuclease, phage-type/RecB, C-terminal | IPR011604 | Phage-Associated | 0 | 119 |
| group_533 | Domain of unknown function DUF4942 | IPR031339 | Other | 0 | 119 |
| group_2670 | Ribbon-helix-helix | IPR010985 | Other | 0 | 119 |
| group_1109 | - | - | Hypothetical Protein | 0 | 119 |
| group_2495 | - | - | Hypothetical Protein | 0 | 119 |
| group_2080 | DinI-like superfamily | IPR036687 | Other | 0 | 118 |
| group_2542 | CblD-like pilus biogenesis initiator | IPR010888 | Other | 0 | 118 |
| group_3358 | Bacteriophage T4, Gp38, tail fibre assembly | IPR003458 | Phage-Associated | 0 | 118 |
| group_2783 | Protein of unknown function DUF1133 | IPR010557 | Other | 0 | 117 |
| group_3283 | Protein of unknown function DUF1367 | IPR009797 | Other | 0 | 116 |
| tfaE/tfaE_1 | - | IPR003458 | Phage-Associated | 0 | 116 |
| group_3455 | Bacteriophage 82, GpQ | IPR010455 | Phage-Associated | 0 | 116 |
| group_3102 | - | - | Hypothetical Protein | 0 | 115 |
| group_2746 | - | - | Hypothetical Protein | 0 | 115 |
| group_281 | Domain of unknown function DUF550 | IPR007538 | Other | 0 | 115 |
| group_1505 | Terminase, large subunit gp17-like | IPR035421 | Phage-Associated | 0 | 115 |
| group_1482 | - | - | Hypothetical Protein | 0 | 115 |
| group_2816 | - | - | Other | 0 | 115 |
| group_1836 | - | - | Hypothetical Protein | 0 | 115 |
| group_2906 | - | - | Hypothetical Protein | 0 | 115 |
| group_2609 | S-adenosyl-L-methionine-dependent methyltransferase | IPR029063 | Other | 0 | 115 |
| group_2616 | - | - | Hypothetical Protein | 0 | 115 |
| group_558 | Abortive infection protein-like, C-terminal domain | IPR026001 | Other | 0 | 115 |
| group_1111 | - | - | Hypothetical Protein | 0 | 115 |
| group_1370 | - | - | Hypothetical Protein | 0 | 115 |
| group_2779 | - | - | Hypothetical Protein | 0 | 115 |
| group_905 | - | - | Hypothetical Protein | 0 | 115 |
| group_2408 | - | - | Hypothetical Protein | 0 | 115 |
| group_3444 | - | - | Hypothetical Protein | 0 | 114 |
| group_3379 | Ead/Ea22-like protein | IPR025153 | Phage-Associated | 0 | 114 |
| group_2222 | - | - | Hypothetical Protein | 0 | 114 |
| group_2502 | KilA, N-terminal | IPR017880 | Other | 0 | 114 |
| group_1708 | - | - | Hypothetical Protein | 0 | 113 |
| group_1559 | Bacteriophage lambda, GpS, holin | IPR006481 | Phage-Associated | 0 | 113 |
| group_3353 | Guanine nucleotide exchange factor SopE | IPR005414 | Virulence-Associated | 0 | 113 |
| group_365 | - | - | Hypothetical Protein | 0 | 112 |

*^a^*Gene identified by Panaroo annotation

*^b^*Genes without a “-” did not have annotations identified by InterPro or IPR and therefore these are categorized as “Hypothetical Protein”

*^c^*IPR entry number assigned by InterPro; for some genes multiple InterPro entry numbers were assigned. For those genes, we reported IPR numbers that were reported the highest number of times, or IPR numbers associated with annotations that represented more specific identifications.

*^d^*Genes were categorized manually based on InterPro annotation.
