# Supplementary material for: Phylogeographic Clustering Suggests that Distinct Clades of Salmonella enterica Serovar Mississippi Are Endemic in Australia, the United Kingdom, and the United States
Source: mSphere. 2021 Sep 22;6(5):e00485-21. doi: 10.1128/mSphere.00485-21 (PMC8550085; doi:10.1128/mSphere.00485-21)
Supplement: TABLE S2 [file msphere.00485-21-st002.docx]

| **Phylogenetic Clade** | **Mississippi Assembly*^a^*** | **Prophage*^b^*** | **Prophage Status*^c^*** | **No. Proteins Detected by Phaster** |
| --- | --- | --- | --- | --- |
| Ai | GCA_008436485 | Gifsy_1 | questionable | 13 |
| Ai | GCA_008436485 | Gifsy_1 | incomplete | 29 |
| Ai | GCA_008436485 | Gifsy_2 | incomplete | 11 |
| Ai | GCA_008436485 | Gifsy_2 | intact | 21 |
| Ai | GCA_008436485 | Salmon_118970_sal3 | incomplete | 33 |
| Ai | GCA_008436485 | Salmon_Fels_1 | incomplete | 7 |
| Ai | GCA_008436485 | Salmon_SEN34 | intact | 65 |
| Ai | GCA_008436485 | Salmon_SJ46 | incomplete | 14 |
| Ai | GCA_008436485 | Salmon_vB_SosS_Oslo | intact | 71 |
| Ai | GCA_008530975 | Gifsy_1 | questionable | 29 |
| Ai | GCA_008530975 | Gifsy_1 | incomplete | 29 |
| Ai | GCA_008530975 | Gifsy_2 | incomplete | 11 |
| Ai | GCA_008530975 | Gifsy_2 | intact | 21 |
| Ai | GCA_008530975 | Salmon_118970_sal3 | incomplete | 27 |
| Ai | GCA_008530975 | Salmon_Fels_1 | incomplete | 7 |
| Ai | GCA_008530975 | Salmon_SEN34 | intact | 61 |
| Ai | GCA_008530975 | Salmon_SJ46 | incomplete | 14 |
| Ai | GCA_008530975 | Salmon_vB_SosS_Oslo | intact | 71 |
| Ai | SRR5921414 | Salmon_Fels_1 | incomplete | 7 |
| Ai | SRR5921414 | Gifsy_1 | incomplete | 29 |
| Ai | SRR5921414 | Gifsy_1 | questionable | 29 |
| Ai | SRR5921414 | Gifsy_2 | intact | 21 |
| Ai | SRR5921414 | Gifsy_2 | incomplete | 11 |
| Ai | SRR5921414 | Haemop_HP1 | intact | 41 |
| Ai | SRR5921414 | Salmon_118970_sal3 | incomplete | 33 |
| Ai | SRR5921414 | Salmon_SEN34 | intact | 61 |
| Ai | SRR5921414 | Salmon_SJ46 | incomplete | 14 |
| Ai | SRR5921414 | Salmon_vB_SosS_Oslo | intact | 71 |
| Ai | SRR5927279 | Gifsy_1 | incomplete | 29 |
| Ai | SRR5927279 | Gifsy_1 | questionable | 29 |
| Ai | SRR5927279 | Gifsy_2 | intact | 21 |
| Ai | SRR5927279 | Gifsy_2 | incomplete | 11 |
| Ai | SRR5927279 | Salmon_118970_sal3 | incomplete | 33 |
| Ai | SRR5927279 | Salmon_Fels_1 | incomplete | 7 |
| Ai | SRR5927279 | Salmon_SEN34 | intact | 65 |
| Ai | SRR5927279 | Salmon_SJ46 | incomplete | 14 |
| Ai | SRR5927279 | Salmon_vB_SosS_Oslo | intact | 71 |
| Ai | SRR5927286 | Entero_ES18 | questionable | 67 |
| Ai | SRR5927286 | Entero_HK630 | incomplete | 10 |
| Ai | SRR5927286 | Gifsy_1 | incomplete | 29 |
| Ai | SRR5927286 | Gifsy_1 | questionable | 29 |
| Ai | SRR5927286 | Gifsy_1 | incomplete | 10 |
| Ai | SRR5927286 | Gifsy_2 | intact | 20 |
| Ai | SRR5927286 | Gifsy_2 | incomplete | 11 |
| Ai | SRR5927286 | Salmon_118970_sal3 | intact | 61 |
| Ai | SRR5927286 | Salmon_Fels_1 | incomplete | 7 |
| Ai | SRR5927286 | Salmon_SEN34 | intact | 60 |
| Ai | SRR5927286 | Salmon_SJ46 | incomplete | 14 |
| Aii | GCA_008526445 | Entero_HK630 | incomplete | 10 |
| Aii | GCA_008526445 | Entero_lambda | incomplete | 22 |
| Aii | GCA_008526445 | Entero_mEp390 | intact | 22 |
| Aii | GCA_008526445 | Entero_mEp460 | intact | 53 |
| Aii | GCA_008526445 | Entero_PsP3 | intact | 48 |
| Aii | GCA_008526445 | Escher_HK639 | incomplete | 20 |
| Aii | GCA_008526445 | Gifsy_1 | incomplete | 12 |
| Aii | GCA_008526445 | Gifsy_2 | incomplete | 12 |
| Aii | GCA_008526445 | Pseudo_PPpW_3 | intact | 53 |
| Aii | GCA_008526445 | Salmon_SEN34 | incomplete | 20 |
| Aii | GCA_008526445 | Salmon_SJ46 | incomplete | 8 |
| Aii | GCA_008588825 | Edward_GF_2 | intact | 52 |
| Aii | GCA_008588825 | Entero_lambda | incomplete | 22 |
| Aii | GCA_008588825 | Entero_mEp237 | incomplete | 16 |
| Aii | GCA_008588825 | Gifsy_1 | intact | 60 |
| Aii | GCA_008588825 | Gifsy_2 | incomplete | 8 |
| Aii | GCA_008588825 | Salmon_118970_sal3 | intact | 49 |
| Aii | GCA_008588825 | Salmon_118970_sal3 | intact | 50 |
| Aii | GCA_008588825 | Salmon_118970_sal3 | incomplete | 10 |
| Aii | GCA_008588825 | Salmon_118970_sal3 | incomplete | 11 |
| Aii | GCA_008588825 | Salmon_Fels_2 | incomplete | 14 |
| Aii | GCA_008588825 | Salmon_SEN34 | intact | 35 |
| Aii | GCA_008588825 | Salmon_SJ46 | incomplete | 8 |
| Aii | GCA_008588825 | Salmon_SPN3UB | incomplete | 31 |
| Aii | GCA_008588845 | Edward_GF_2 | intact | 51 |
| Aii | GCA_008588845 | Entero_lambda | incomplete | 22 |
| Aii | GCA_008588845 | Entero_mEp237 | incomplete | 16 |
| Aii | GCA_008588845 | Entero_mEp460 | intact | 59 |
| Aii | GCA_008588845 | Gifsy_1 | intact | 61 |
| Aii | GCA_008588845 | Salmon_118970_sal3 | intact | 49 |
| Aii | GCA_008588845 | Salmon_118970_sal3 | incomplete | 10 |
| Aii | GCA_008588845 | Salmon_118970_sal3 | intact | 51 |
| Aii | GCA_008588845 | Salmon_118970_sal3 | incomplete | 13 |
| Aii | GCA_008588845 | Salmon_Fels_2 | incomplete | 17 |
| Aii | GCA_008588845 | Salmon_SEN34 | intact | 63 |
| Aii | GCA_008588845 | Salmon_SJ46 | incomplete | 8 |
| Aii | GCA_008591505 | Edward_GF_2 | intact | 53 |
| Aii | GCA_008591505 | Entero_lambda | incomplete | 22 |
| Aii | GCA_008591505 | Entero_mEp237 | incomplete | 16 |
| Aii | GCA_008591505 | Entero_mEp460 | intact | 59 |
| Aii | GCA_008591505 | Gifsy_1 | intact | 60 |
| Aii | GCA_008591505 | Gifsy_2 | incomplete | 8 |
| Aii | GCA_008591505 | Salmon_118970_sal3 | intact | 51 |
| Aii | GCA_008591505 | Salmon_118970_sal3 | intact | 49 |
| Aii | GCA_008591505 | Salmon_118970_sal3 | incomplete | 13 |
| Aii | GCA_008591505 | Salmon_118970_sal3 | incomplete | 10 |
| Aii | GCA_008591505 | Salmon_Fels_2 | incomplete | 17 |
| Aii | GCA_008591505 | Salmon_SEN34 | intact | 66 |
| Aii | GCA_008591505 | Salmon_SJ46 | incomplete | 8 |
| Aii | GCA_008591565 | Edward_GF_2 | intact | 52 |
| Aii | GCA_008591565 | Entero_lambda | incomplete | 22 |
| Aii | GCA_008591565 | Entero_mEp237 | incomplete | 16 |
| Aii | GCA_008591565 | Gifsy_1 | intact | 22 |
| Aii | GCA_008591565 | Gifsy_2 | incomplete | 8 |
| Aii | GCA_008591565 | Gifsy_2 | questionable | 19 |
| Aii | GCA_008591565 | Salmon_118970_sal3 | intact | 51 |
| Aii | GCA_008591565 | Salmon_118970_sal3 | intact | 49 |
| Aii | GCA_008591565 | Salmon_118970_sal3 | incomplete | 13 |
| Aii | GCA_008591565 | Salmon_118970_sal3 | incomplete | 10 |
| Aii | GCA_008591565 | Salmon_Fels_2 | incomplete | 18 |
| Aii | GCA_008591565 | Salmon_SEN34 | incomplete | 38 |
| Aii | GCA_008591565 | Salmon_SEN34 | intact | 66 |
| Aii | GCA_008591565 | Salmon_SJ46 | incomplete | 8 |

*^a^*Mississippi assembly queried; each line represents a different prophage region detected by Phaster

*^b^*Prophage identity reported represents the top hit provided by Phaster

*^c^*Prophage status represents the score given by Phaster. Prophage genomes are rated as intact, questionable, or incomplete based on scoring criteria that considers the number of phage coding sequences detected, the presence of specific prophage terms (e.g., capsid, tail, head, etc.), and the number of hypothetical proteins in the identified prophage region.
