# Supplementary material for: Phylogeographic Clustering Suggests that Distinct Clades of Salmonella enterica Serovar Mississippi Are Endemic in Australia, the United Kingdom, and the United States
Source: mSphere. 2021 Sep 22;6(5):e00485-21. doi: 10.1128/mSphere.00485-21 (PMC8550085; doi:10.1128/mSphere.00485-21)
Supplement: TABLE S3 [file msphere.00485-21-st003.docx]

| **Gene*^a^*** | **InterPro Annotation*^b^*** | **IPR No.*^b,c^*** | **Category*^d^*** | **No. Bi Genomes with Gene** | **No. Bii Genomes with Gene** |
| --- | --- | --- | --- | --- | --- |
| group_484 | Conserved hypothetical protein CHP01619 | IPR006506 | Other | 134 | 0 |
| group_487 | GIY-YIG endonuclease | IPR000305 | Other | 134 | 0 |
| group_808 | - | - | Hypothetical Protein | 134 | 0 |
| group_957 | - | - | Hypothetical Protein | 133 | 0 |
| group_920 | - | - | Hypothetical Protein | 133 | 0 |
| group_844 | - | - | Hypothetical Protein | 133 | 0 |
| group_852 | - | - | Hypothetical Protein | 133 | 0 |
| group_1024 | Immunity protein 8 | IPR028964 | Other | 133 | 0 |
| group_984 | - | - | Hypothetical Protein | 133 | 0 |
| rhsC_3 | RHS protein | IPR001826 | Other | 133 | 0 |
| group_518 | Protein of unknown function DUF1120 | IPR010546 | Other | 133 | 0 |
| group_517 | Protein of unknown function DUF1120 | IPR010546 | Other | 133 | 0 |
| yraJ | Outer membrane usher protein | IPR000015 | Virulence-associated | 133 | 0 |
| yraI | Pili assembly chaperone, bacterial | IPR001829 | Virulence-associated | 133 | 0 |
| symE_2 | Toxin SymE-like | IPR014944 | Other | 133 | 0 |
| group_986 | Transposase, IS4-like | IPR002559 | Integrative Conjugative Element | 133 | 0 |
| group_371 | - | - | Hypothetical Protein | 133 | 0 |
| yehB_1 | Outer membrane usher protein | IPR000015 | Virulence-associated | 132 | 0 |
| aes_1 | Alpha/beta hydrolase fold-3 | IPR013094 | Other | 131 | 0 |
| sopD_2 | Salmonella outer protein D | IPR022747 | Virulence-associated | 130 | 0 |
| dnaQ_1 | Ribonuclease H-like superfamily | IPR012337 | Other | 0 | 7 |
| group_278 | Conjugative transfer ATPase | IPR022303 | Integrative Conjugative Element | 0 | 7 |
| group_284 | Type IV secretion system coupling protein TraD DNA-binding domain | IPR019476 | Integrative Conjugative Element | 0 | 7 |
| group_292 | ParB/Sulfiredoxin | IPR003115 | Integrative Conjugative Element | 0 | 7 |
| group_295 | TraG-like, N-terminal | IPR012931 | Integrative Conjugative Element | 0 | 7 |
| group_299 | Integrating conjugative element protein, PFL4705 | IPR021207 | Integrative Conjugative Element | 0 | 7 |
| group_301 | Toll/interleukin-1 receptor homology (TIR) domain | IPR000157 | Other | 0 | 7 |
| group_302 | Integrating conjugative element protein, PFL4711 | IPR021204 | Integrative Conjugative Element | 0 | 7 |
| group_308 | Putative conjugal transfer nickase/helicase TraI, C-terminal | IPR011093 | Integrative Conjugative Element | 0 | 7 |
| group_312 | DNA helicase, DnaB type | IPR007692 | Integrative Conjugative Element | 0 | 7 |
| group_335 | - | - | Hypothetical Protein | 0 | 7 |
| group_375 | Protein of unknown function DUF1963 | IPR015315 | Other | 0 | 7 |
| group_416 | Protein of unknown function DUF1202 | IPR009592 | Other | 0 | 7 |
| group_431 | Abortive infection protein-like, C-terminal domain | IPR026001 | Other | 0 | 7 |
| group_436 | Integrase, catalytic domain | IPR002104 | Integrative Conjugative Element | 0 | 7 |
| group_440 | TraU | IPR009649 | Integrative Conjugative Element | 0 | 7 |
| group_456 | - | - | Hypothetical Protein | 0 | 7 |
| group_464 | Integrating conjugative element protein, PFL4704 | IPR021844 | Integrative Conjugative Element | 0 | 7 |
| group_465 | AAA domain | IPR025669 | Other | 0 | 7 |
| group_491 | Integrating conjugative element protein, PFL4697-type | IPR022266 | Integrative Conjugative Element | 0 | 7 |
| group_492 | Integrating conjugative element protein, PFL4693 | IPR022293 | Integrative Conjugative Element | 0 | 7 |
| group_496 | Integrating conjugative element protein, PFL4669 | IPR014996 | Integrative Conjugative Element | 0 | 7 |
| group_506 | - | - | Hypothetical Protein | 0 | 7 |
| group_536 | Protein of unknown function DUF2895 | IPR021548 | Other | 0 | 7 |
| group_553 | Integrating conjugative element protein PilL, PFGI-1 | IPR022260 | Integrative Conjugative Element | 0 | 7 |
| group_556 | - | - | Hypothetical Protein | 0 | 7 |
| group_565 | Transglycosylase SLT domain 1 | IPR008258 | Other | 0 | 7 |
| group_569 | - | - | Hypothetical Protein | 0 | 7 |
| group_580 | Protein of unknown function DUF3577 | IPR021960 | Other | 0 | 7 |
| group_584 | Protein of unknown function DUF2857 | IPR021364 | Other | 0 | 7 |
| group_594 | Integrating conjugative element protein, PFL4695 | IPR021300 | Integrative Conjugative Element | 0 | 7 |
| group_649 | - | - | Hypothetical Protein | 0 | 7 |
| group_656 | Lipoprotein, putative | IPR022262 | Other | 0 | 7 |
| group_658 | Integrating conjugative element protein, PFL4709 | IPR011090 | Integrative Conjugative Element | 0 | 7 |
| group_665 | - | - | Hypothetical Protein | 0 | 7 |
| group_668 | - | - | Hypothetical Protein | 0 | 7 |
| group_670 | Protein of unknown function DUF3487 | IPR021877 | Other | 0 | 7 |
| group_673 | Integrating conjugative element protein PFL4702 | IPR021356 | Integrative Conjugative Element | 0 | 7 |
| group_692 | - | - | Hypothetical Protein | 0 | 7 |
| group_696 | - | - | Hypothetical Protein | 0 | 7 |
| group_709 | Uncharacterised protein family, RAQPRD | IPR019110 | Other | 0 | 7 |
| group_717 | - | - | Hypothetical Protein | 0 | 7 |
| group_730 | - | - | Hypothetical Protein | 0 | 7 |
| group_735 | - | - | Hypothetical Protein | 0 | 7 |
| group_744 | - | - | Hypothetical Protein | 0 | 7 |
| group_755 | - | - | Hypothetical Protein | 0 | 7 |
| group_766 | Zinc finger, DksA/TraR C4-type | IPR000962 | Integrative Conjugative Element | 0 | 7 |
| group_771 | Protein of unknown function DUF3262 | IPR021676 | Other | 0 | 7 |
| group_795 | - | - | Hypothetical Protein | 0 | 7 |
| group_814 | - | - | Hypothetical Protein | 0 | 7 |
| group_816 | - | - | Hypothetical Protein | 0 | 7 |
| group_824 | - | - | Hypothetical Protein | 0 | 7 |
| klcA | Antirestriction protein | IPR004914 | Integrative Conjugative Element | 0 | 7 |
| topB_2 | DNA topoisomerase, type IA, domain 2 | IPR003601 | Integrative Conjugative Element | 0 | 7 |
| traC | Domain of unknown function DUF1738 | IPR013610 | Integrative Conjugative Element | 0 | 7 |
| xerC_2 | Integrase/recombinase, N-terminal | IPR010998 | Integrative Conjugative Element | 0 | 7 |
| yadA | YadA-like, C-terminal | IPR005594 | Virulence-associated | 0 | 7 |

*^a^*Gene identified by Panaroo annotation

*^b^*Genes without a “-” did not have annotations identified by InterPro or IPR and therefore these are categorized as “Hypothetical Protein”

*^c^*IPR entry number assigned by InterPro; for some genes multiple InterPro entry numbers were assigned. For those genes, we reported IPR numbers that were reported the highest number of times, or IPR numbers associated with annotations that represented more specific identifications.

*^d^*Genes were categorized manually based on InterPro annotation.
