# Supplementary material for: Phylogeographic Clustering Suggests that Distinct Clades of Salmonella enterica Serovar Mississippi Are Endemic in Australia, the United Kingdom, and the United States
Source: mSphere. 2021 Sep 22;6(5):e00485-21. doi: 10.1128/mSphere.00485-21 (PMC8550085; doi:10.1128/mSphere.00485-21)
Supplement: TABLE S4 [file msphere.00485-21-st004.docx]

| ***S.* Mississippi Clade** | **Total # isolates** | ***wcmC*** | |  | ***wfbI*** | |  | ***wzx*** | |  | ***wzy*** | | |
| --- | --- | --- | --- | --- | --- | --- | --- | --- | --- | --- | --- | --- | --- |
|  |  | **No. with full-length*^a^*** | **No. with mismatches (# SNPs)*^b,c^*** |  | **No. with full-length** | **No. with mismatches**  **(# SNPs)** |  | **No. with full-length** | **No. with mismatches (# SNPs)** |  | **No. with full-length** | **No. with mismatches (# SNPs)** |  |
| Ai | 99 | 85 | 1 (0-1) |  | 96 | 1 (0-1) |  | 74 | 4 (0-1) |  | 57 | 12 (0-2) |  |
| Aii | 124 | 95 | 94 (0-1) |  | 123 | 123 (1-3) |  | 78 | 2 (0-1) |  | 28 | 1 (0-1) |  |
| Bi | 134 | 121 | 0 (NA) |  | 132 | 4 (0-1) |  | 113 | 0 (NA) |  | 80 | 4 (0-1) |  |
| Bii | 7 | 6 | 0 (NA) |  | 7 | 0 (NA) |  | 5 | 0 (NA) |  | 4 | 0 (NA) |  |

*^a^*The number of isolates in the clade that have the full-length gene product in the blastn results. Using this stringent cut-off, only hits with 738 nt. for *wfbH*, 861 nt. for *wfbI*, 1236 nt. for *wzx*, or 1167 nt. for *wzy* were counted.

*^b^*Includes the number of isolates in the clade that have at least 1 single nucleotide polymorphism compared to the isolate used as query.

*^c^*numbers in parentheses represent the range of mismatches per isolate. For example, for *wcmC* clade Ai isolates, 85 had a full-length hit detected with blastn, with one isolate having a single SNP; NA: not applicable as none of the isolates had a SNP, compared to the sequence used as query.
