# Supplementary figures and images for: Phylogeographic Clustering Suggests that Distinct Clades of Salmonella enterica Serovar Mississippi Are Endemic in Australia, the United Kingdom, and the United States
Source: mSphere. 2021 Sep 22;6(5):e00485-21. doi: 10.1128/mSphere.00485-21 (PMC8550085; doi:10.1128/mSphere.00485-21)

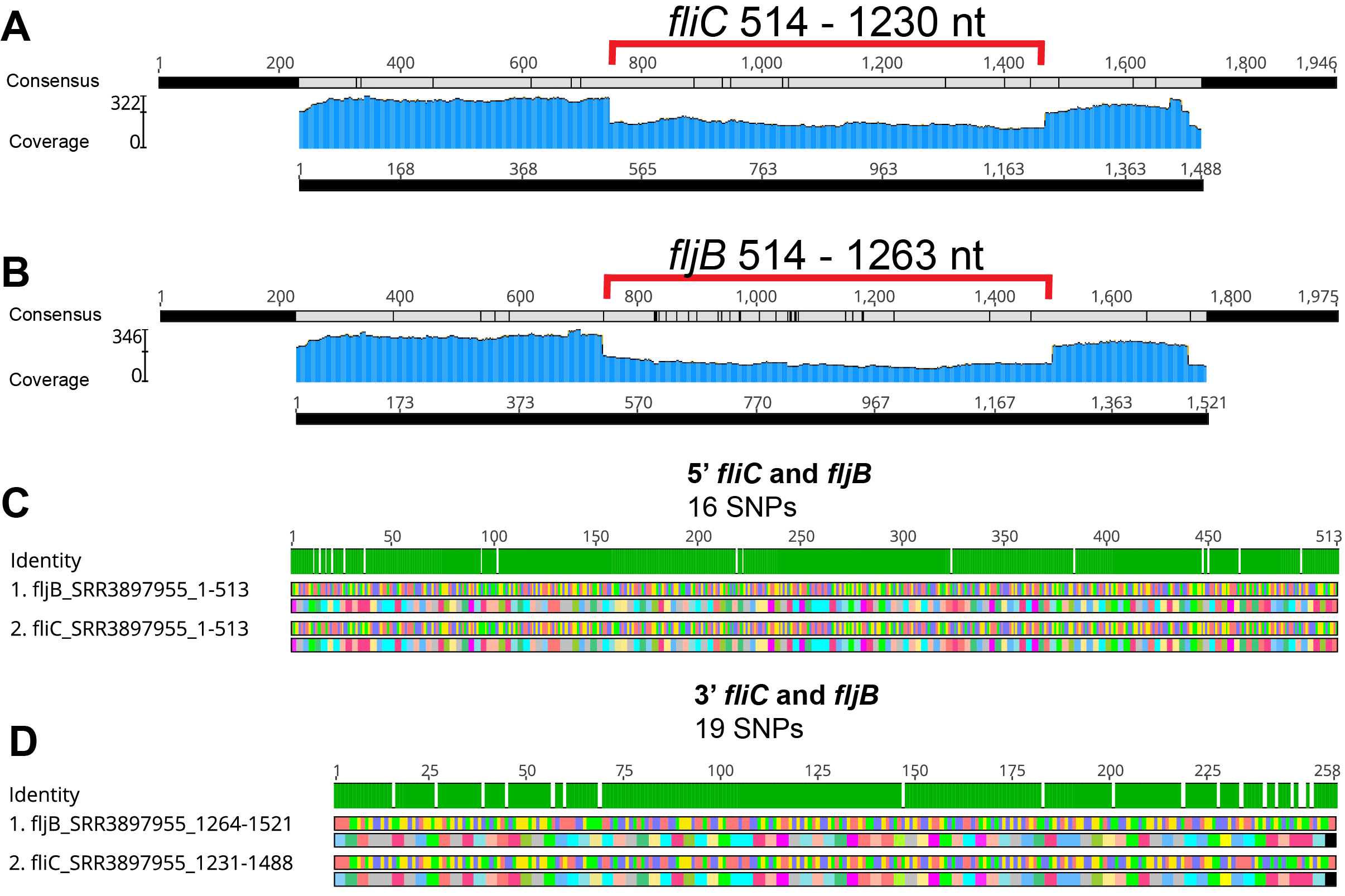

Supplement: FIG S1 [file msphere.00485-21-sf001.tif]
